# Cell-specific microarray profiling experiments reveal a comprehensive picture of gene expression in the *C. elegans *nervous system

**DOI:** 10.1186/gb-2007-8-7-r135

**Published:** 2007-07-05

**Authors:** Stephen E Von Stetina, Joseph D Watson, Rebecca M Fox, Kellen L Olszewski, W Clay Spencer, Peter J Roy, David M Miller

**Affiliations:** 1 Department of Cell and Developmental Biology, Vanderbilt University, Nashville, TN 37232-8240, USA; 2 Graduate Program in Neuroscience, Center for Molecular Neuroscience, Vanderbilt University, Nashville, TN 37232-8548, USA; 3 Department of Cell Biology, Johns Hopkins School of Medicine, Baltimore, MD 21205, USA; 4 Department of Molecular Biology, Lewis-Sigler Institute for Integrative Genomics, Princeton University 246 Carl Icahn Laboratory, Princeton NJ 08544, USA; 5 Department of Medical Genetics and Microbiology, Donnelly Centre for Cellular and Biomolecular Research, University of Toronto, Toronto, ON, M5S 1A, Canada

## Abstract

**Background:**

With its fully sequenced genome and simple, well-defined nervous system, the nematode *Caenorhabditis elegans *offers a unique opportunity to correlate gene expression with neuronal differentiation. The lineal origin, cellular morphology and synaptic connectivity of each of the 302 neurons are known. In many instances, specific behaviors can be attributed to particular neurons or circuits. Here we describe microarray-based methods that monitor gene expression in *C. elegans *neurons and, thereby, link comprehensive profiles of neuronal transcription to key developmental and functional properties of the nervous system.

**Results:**

We employed complementary microarray-based strategies to profile gene expression in the embryonic and larval nervous systems. In the MAPCeL (Microarray Profiling *C. elegans *cells) method, we used fluorescence activated cell sorting (FACS) to isolate GFP-tagged embryonic neurons for microarray analysis. To profile the larval nervous system, we used the mRNA-tagging technique in which an epitope-labeled mRNA binding protein (FLAG-PAB-1) was transgenically expressed in neurons for immunoprecipitation of cell-specific transcripts. These combined approaches identified approximately 2,500 mRNAs that are highly enriched in either the embryonic or larval *C. elegans *nervous system. These data are validated in part by the detection of gene classes (for example, transcription factors, ion channels, synaptic vesicle components) with established roles in neuronal development or function. Of particular interest are 19 conserved transcripts of unknown function that are also expressed in the mammalian brain. In addition to utilizing these profiling approaches to define stage-specific gene expression, we also applied the mRNA-tagging method to fingerprint a specific neuron type, the A-class group of cholinergic motor neurons, during early larval development. A comparison of these data to a MAPCeL profile of embryonic A-class motor neurons identified genes with common functions in both types of A-class motor neurons as well as transcripts with roles specific to each motor neuron type.

**Conclusion:**

We describe microarray-based strategies for generating expression profiles of embryonic and larval *C. elegans *neurons. These methods can be applied to particular neurons at specific developmental stages and, therefore, provide an unprecedented opportunity to obtain spatially and temporally defined snapshots of gene expression in a simple model nervous system.

## Abstract 2

A novel strategy for profiling *Caenorhabditis elegans *cells identifies transcripts highly enriched in either the embryonic or larval *C. elegans *nervous system, including 19 conserved transcripts of unknown function that are also expressed in the mammalian brain.

## Background

**Figure 1:**
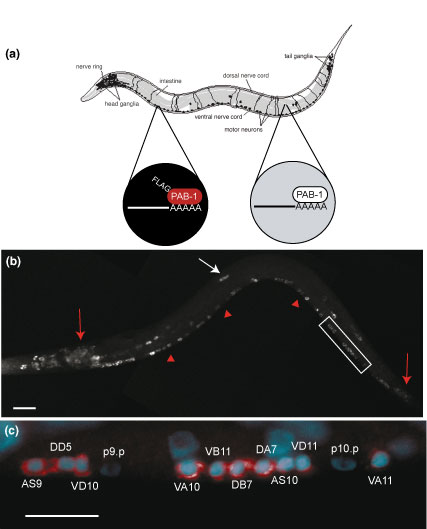
mRNA-tagging isolates neural specific transcripts. **(a) **The mRNA-tagging strategy for profiling gene expression in the *C. elegans *nervous system. A pan-neural promoter drives expression of FLAG-tagged poly-A binding protein (*F25B3.3*::FLAG-PAB-1) in neurons (black). Native PAB-1 is ubiquitously expressed in all cells (gray). Neural-specific transcripts are isolated by coimmunoprecipitation with anti-FLAG antibodies (artwork courtesy of Erik Jorgensen). **(b) **Immunostaining detects FLAG::PAB-1 expression in neurons in head and tail ganglia (red arrows), ventral nerve cord motor neurons (red arrowheads), and touch neurons (white arrow). Lateral view of L2 larvae. Anterior to left. **(c) **Close-up view of posterior ventral cord (boxed area in (b)), showing anti-FLAG staining (red) in cytoplasm surrounding motor neuron nuclei (for example, AS9, DD5, and so on) stained with DAPI (blue). Note that hypodermal blast cells (P9p and P10p) do not show anti-FLAG staining. Anterior is left, ventral is down. Scale bars = 10 μm.

The nematode *Caenorhabditis elegans *is a widely used model system for developmental studies. The major tissues of complex metazoans, (muscle, intestine, nervous system, skin, and so on) are represented in the worm, but the entire animal is composed of fewer than 1,000 somatic cells. Owing to this simplicity and to the rapid development of the *C. elegans *body plan, the anatomy of every adult cell has been described and the patterns of division giving rise to each one are known [1,2]. The *C. elegans *genome is fully sequenced [3,4] and encodes over 20,000 predicted genes. Thus, *C. elegans *offers a unique opportunity to identify specific combinations of genes that define the differentiation and structure of specific cell types. In principle, microarray profiles can provide this information. In order to implement this strategy, however, the small size of *C. elegans *(length = 1 mm) has required the development of specialized methods for extracting mRNA from specific cell types. In one approach, MAPCeL (micro-array profiling of *C. elegans *cells), green-fluorescent protein (GFP)labeled cells are isolated by fluorescence activated cell sorting (FACS) from preparations of dissociated embryonic cells [5]. This method has now been used to profile global gene expression in specific subsets of neurons and muscle cells [5-10] (RMF, DMM, unpublished data). An alternative technique, mRNA-tagging [11], can be utilized to profile larval cells, which are not readily accessible for FACS [12]. In this approach, an epitopetagged mRNA binding protein (FLAG-PAB) is expressed transgenically with a specific promoter (Figure 1). FLAG-PAB-bound transcripts are then immunoprecipitated for microarray analysis. mRNA-tagging profiles have been reported for two major tissues, body wall muscles and the intestine [11,13].

Here we apply the MAPCeL and mRNA-tagging strategies to provide a comprehensive picture of gene expression in the embryonic and larval nervous systems. This analysis reveals approximately 2,500 transcripts that are significantly elevated in neurons versus other *C. elegans *cell types during these developmental periods. The enrichment in these datasets of transcripts known to be expressed in neurons, as well as newly created GFP reporters from previously uncharacterized genes in these lists, confirmed the tissue specificity of our results. The 'pan-neural' transcripts detected in these datasets encode proteins with a wide array of molecular functions, including ion channels, neurotransmitter receptors and transcription factors. Overall, 56% of these *C. elegans *genes are conserved in humans. The discovery of 27 uncharacterized human homologs enriched in both embryonic and larval neurons suggests that these profiles have uncovered novel genes with potentially conserved function in the nervous system.

In order to identify transcripts that are selectively expressed in a specific neural cell type, we used the mRNA-tagging strategy to fingerprint a subset of motor neurons (A-class) in the ventral nerve cord of L2 stage larvae. This A-class dataset contains around 400 significantly enriched genes. Approximately 25% of these transcripts are not detected in the profile of the entire nervous system. This finding suggests that individual neurons may express rare transcripts that are likely to be restricted to specific neuron types. The application of the mRNA-tagging strategy to profile a specific class of larval neurons complements earlier work in which this method was used to profile larval ciliated neurons [14] and also experiments in which MAPCeL and other FACS-based approaches have been applied to selected embryonic neurons [5-10]. Thus, this work demonstrates the utility of complementary profiling strategies that can now be applied to catalog gene expression in specific *C. elegans *neurons throughout development.

## Results

### Neuronal mRNA-tagging yields reproducible microarray expression profiles

To profile gene expression throughout the nervous system, we generated a stable, chromosomally integrated transgenic line expressing an epitope-tagged poly-A binding protein (FLAG::PAB-1) throughout the nervous system. Pan-neuronal expression was confirmed by immunostaining with a FLAG-specific antibody (Figure 1). We selected the second larval stage (L2) to test the application of the mRNA-tagging method. At this stage, the nervous system is largely in place and should, therefore, express a broad array of transcripts that define the development and function of most neurons. Sub-microgram quantities of mRNA isolated by the mRNA-tagging method were amplified and labeled for application to an Affymetrix chip representing approximately 90% of predicted *C. elegans *genes. Neuron-enriched transcripts in these samples were detected by comparison to a reference profile of all larval cells (see Materials and methods). We reasoned that this approach should detect a significant fraction of known neuronal transcripts and thus provide an initial test of the specificity of this strategy.

**Figure 2:**
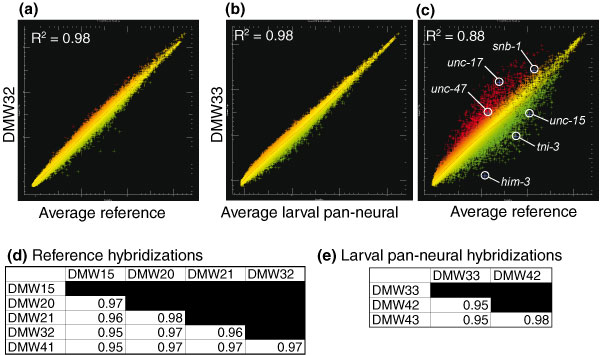
Microarray profiles reveal transcripts enriched in *C. elegans *neurons. **(a) **Scatter plot of intensity values (log base 2) for representative hybridization (DMW32; red) of RNA isolated from all larval cells (reference) by mRNA-tagging compared to the average intensity of the reference dataset (green). **(b) **Scatter plot of a representative larval pan-neural hybridization (DMW33; red) compared to the average intensities for all three larval pan-neural hybridizations (green). **(c) **Results of a single larval pan-neural hybridization (DMW33; red) compared to average reference intensities (green) to identify differentially expressed transcripts. Known neural genes *snb-1 *(synaptobrevin, all neurons), *unc-17 *(VAChT, cholinergic neurons), and *unc-47 *(VGAT, GABAergic neurons) are enriched (red). Depleted genes include two muscle-specific transcripts (*unc-15*, paramyosin, and *tni-3*, troponin) and a germline-specific gene (*him-3*) (green). **(d,e) **Pairwise comparisons of individual hybridizations. Coefficient of determination (R^2^) values for all pairwise combinations of reference hybridizations (d) and for all pairwise combinations of larval pan-neural hybridizations (e) indicate reproducible results for both reference and experimental samples.

Comparisons of independently derived datasets for both the experimental (larval pan-neural) and reference samples showed that individual replicates for each condition are highly reproducible (Figure 2a,b). For example, an average coefficient of determination (R^2^) of approximately 0.96 was calculated from pairwise combinations of each individual reference dataset (Figure 2d). The pan-neural datasets were similarly reproducible (R^2 ^of approximately 0.96; Figure 2e). The overall concurrence of these data is graphically illustrated in the scatter plots shown in Figure 2a,b.

### Transcripts detected by neuronal mRNA-tagging are expressed in neurons

**Figure 3:**
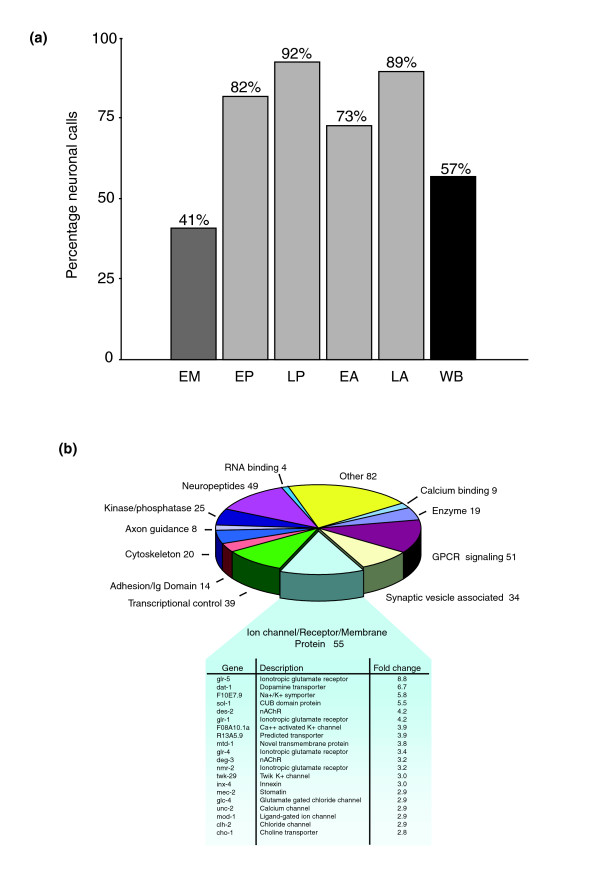
Microarray profiles detect known *C. elegans *neural genes. **(a) **Histogram showing fraction of annotated genes in microarray datasets with known *in vivo *expression in neurons. The list of annotated genes used for this comparison includes all genes with known cellular expression patterns listed in WormBase (see Materials and methods). Note significant enrichment for neuronal genes in microarray datasets obtained from neurons (73-92%) relative to the fraction of all annotated genes in WormBase (57%) and embryonic muscle (41%) that show some expression in the nervous system. Microarray datasets are: EM, embryonic muscle; EP, embryonic pan-neural; LP, larval pan-neural; EA, embryonic A-class motor neuron; LA, larval A-class motor neuron; WB, WormBase. **(b) **The larval pan-neural enriched dataset contains 443 transcripts previously annotated as expressed in neurons in WormBase. Genes were grouped according to functional categories characteristic of neurons. The top 20 enriched ion channel/receptor/membrane proteins are featured (Additional data file 7).

**Figure 4:**
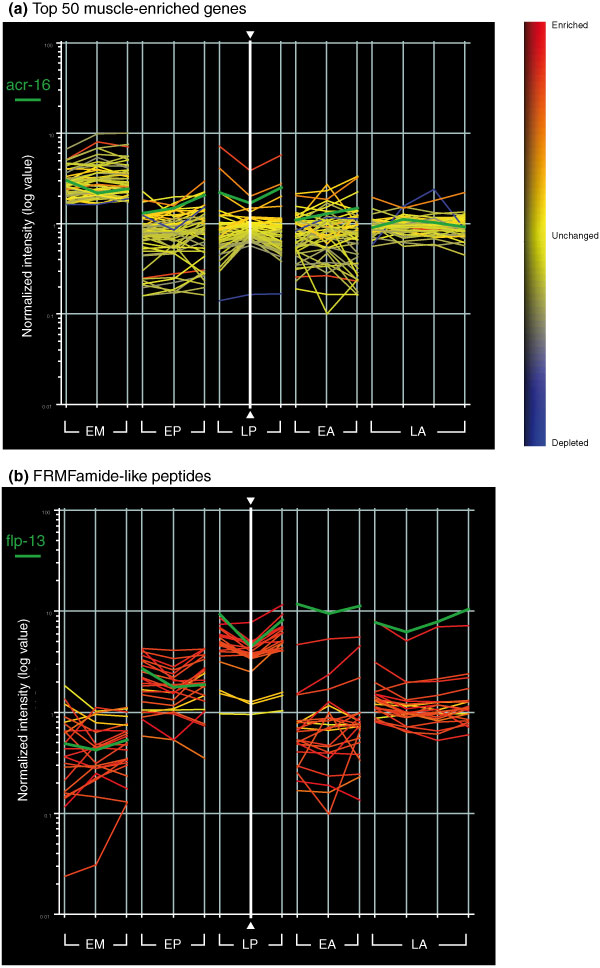
Neuropeptides are highly represented in profiles of neural cells while transcripts highly enriched in body wall muscle are excluded. Line graphs display log base 10 of relative intensity values (experimental/reference) for selected genes on the *C. elegans *Affymetrix array (see Materials and methods). Vertical lines correspond to individual replicates for each experimental sample. Thus, trends in expression levels for a particular gene or sets of genes can be visualized across all datasets. EM, embryonic muscle; EP, embryonic pan-neural; LP, larval pan-neural; EA, embryonic A-class motor neuron; LA, larval A-class motor neuron. Horizontal lines are colored (see heat map at right) according to relative enrichment of a single LP replicate (vertical white line with arrowheads): enriched (red), blue (depleted) and yellow (no change). **(a) **The top-50 ranked genes from embryonic muscle show limited enrichment in neuronal datasets. One exception is *acr-16*, marked by the horizontal green line, which is highly enriched in the LP dataset. *acr-16 *encodes a nicotinic acetylcholine receptor that is expressed in both muscle cells and neurons [16,17]. **(b) **FRMFamide-like peptides (*flp*) are enriched in neurons. A majority (20/23) of the 23 defined *flp *transcripts is enriched in the LP dataset, whereas specific subsets of *flp *transcripts are enriched in other neuronal datasets (EP, EA, LA) but largely excluded from the muscle (EM) dataset. The horizontal green highlights *flp-13*, which is the most highly enriched *flp *transcript in the A-class motor neuron (EA, LA) datasets.

**Figure 5:**
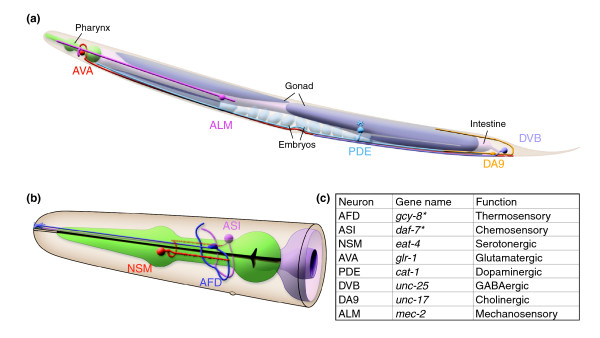
Pan-neural datasets detect neuron-specific transcripts. A representation of transcripts enriched in the larval pan-neural dataset and a subset of the neurons in which these genes are expressed. **(a) **Lateral view of an adult worm depicting selected neurons. Ventral is down, anterior is to the left. **(b) **Close-up of the adult head, showing the serotonergic neuron NSM and two sensory neurons, AFD and ASI. For simplicity, only one of the two pairs of neurons is diagrammed. The pharynx is colored green and the anterior end of the intestine is gray. **(c) **Table displaying representative genes enriched in the larval pan-neural dataset and expressed in each indicated neuron. Asterisks denote exclusive expression in the listed cell type. (Artwork courtesy of Zeynep Altun, Chris Crocker and David Hall at WormAtlas [120].)

Scatter plots comparing larval pan-neural versus reference data revealed a substantial number of transcripts with significant differences in hybridization intensities (Figure 2c). Statistical analysis detected 1,562 transcripts with elevated expression (≥ 1.5-fold, ≤ 1% false discovery rate (FDR)) in the larval pan-neural sample (Additional data file 1). Strikingly, we found that 92% of the 443 genes with known expression patterns included in the larval pan-neural enriched dataset (409/443) are listed in WormBase [15] as neuronally expressed (Figure 3a; Additional data file 1). By contrast, only 57% of all genes (1,612/2,837) with defined expression patterns in WormBase are annotated as expressed in neurons (see Materials and methods; Figure 3a; Additional data files 2 and 3). Moreover, genes with key roles in neuronal function are highly represented in this list. For example, 55 transcripts encoding ion channels, receptors or membrane proteins with known expression in the *C. elegans *nervous system are enriched (Figure 3b; Additional data file 7). The enrichment of transcripts known to be expressed in neurons demonstrates that the larval pan-neural profile is largely derived from neural tissue. This conclusion is also substantiated by the finding that mRNAs highly expressed in other cell types are preferentially excluded from this dataset (Figure 2c). For example, microarray profiling experiments identified a total of 1,926 transcripts enriched in either larval germline, muscle or intestinal cells (GMI; Additional data file 5) [13]. This set of genes is significantly under-represented (97/1,562) in the larval pan-neural dataset (representation factor 0.6, *p *< 2.033e^-9^; a representation factor <1 indicates under-representation; see Materials and methods). Of the 97 genes that intersect our larval pan-neural profile and the GMI set, 35 have a previously characterized spatial expression pattern. Of these, 89% (31/35) are also expressed in neurons. A comparison of the top 50 most significantly enriched transcripts in a MAPCeL profile of embryonic body wall muscle cells (RMF, DMM, unpublished data) detected only four transcripts that also show elevated expression in the larval pan-neural profile (Figure 4a; Additional data file 6). Independent results have confirmed that at least one of these, the acetylcholine receptor subunit *acr-16*, is expressed in both muscle and neurons [16,17]. The apparent low frequency of false positives empirically defined by these comparisons is consistent with the estimated FDR of ≤ 1% for this dataset. The stringent exclusion of non-neuronal transcripts has been achieved, however, while retaining sensitivity to transcripts that may be expressed in limited numbers of neurons (Figure 5). For example, our methodology identifies genes that are expressed in only two neurons; *daf-7 *(transforming growth factor (TGF)-beta-like peptide expressed in ASIL and ASIR) [18] and *gcy-8 *(guanylate cyclase expressed in AFDL and AFDR) [19] (Figure 5).

**Figure 6:**
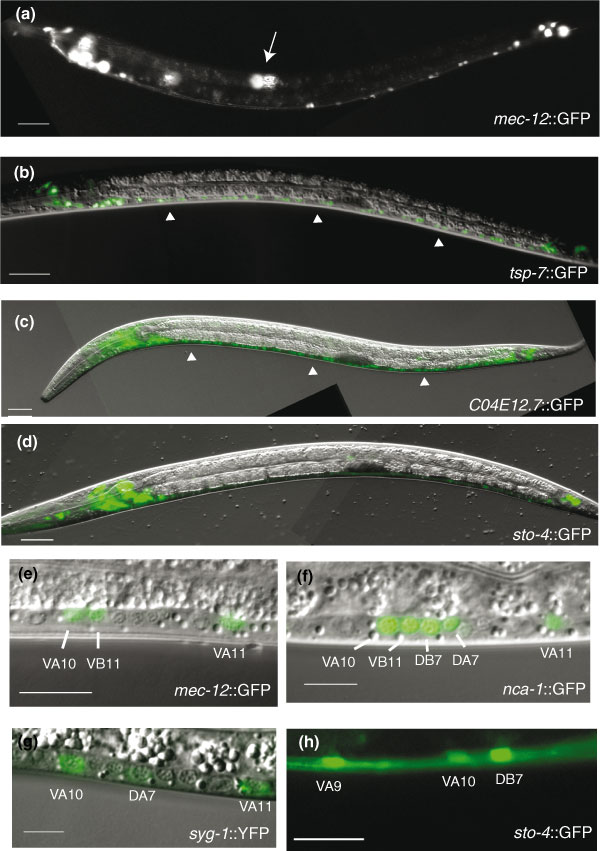
GFP reporters validate neuronal microarray datasets. Transgenic animals expressing GFP reporters for representative genes detected in neuron-enriched microarray datasets. Anterior to left, ventral down. GFP images are combined with matching DIC micrographs for panels (b-g). **(a,e) ***mec-12*::GFP is expressed in touch neurons (arrow) and in specific ventral cord motor neurons (e) at the L2 stage. **(b,c) ***tsp-7*::GFP and *C04E12.7*::GFP are widely expressed in the nervous system with bright GFP in head and tail ganglia and in motor neurons of the ventral nerve cord (arrow heads). **(d,f,g,h) **Note expression of GFP reporters for *sto-4*, *nca-1*, and *syg-1 *in A-class (DA, VA) and in other ventral cord motor neurons (for example, DB, VB).

The strong enrichment of known neuronal genes in the larval pan-neural dataset indicates that other previously uncharacterized transcripts in this list are also likely to be expressed in the nervous system. To test this prediction, we evaluated GFP reporter genes for representative transcripts in this profile. As shown in Table 1 and Additional data file 17, all but one of the transgenic lines (24 of 25) derived from these promoter GFP fusions show expression in neurons (Figure 6). Of the GFP reporters tested, 56% (14/25) are exclusively detected in neurons (Additional data file 17). For example, the stomatin gene *sto-4 *is highly expressed in ventral cord motor neurons, touch neurons and in head and tail ganglia (Table 1; Figure 6d,h). Our GFPreporter analysis demonstrates that the remaining 11 genes tested are expressed in other tissues in addition to neurons. For instance, the GFP reporter for C04E12.7 (phospholipid scramblase), which is expressed widely throughout the nervous system, is also expressed in muscle cells (Table 1; Figure 6c). Thus, these results indicate that the genes identified in the larval pan-neural profile largely fall into two classes; those that are exclusively expressed in neurons, and those that are expressed in multiple tissues, including neurons. Our finding of neuronal GFP expression for transcripts exhibiting a wide range of enrichment (1.5- to 8.3-fold) predicts that most of the genes in this list that have not been directly tested are also likely to be expressed in neurons. Together, these results demonstrate that our pan-neural mRNA-tagging approach enriches for *bona fide *neuronally expressed transcripts and effectively excludes transcripts expressed exclusively in other tissues.

**Table 1:** Expression of promoter-GFP reporters for transcripts enriched in the embryonic pan-neural, larval pan-neural or A-class motor neuron datasets

			Pan-neural			A-class	
				
Cosmid	Gene	Protein	EP fold change	LP fold change	In neurons*	Fold change	UNC-4 neuron(s)*
C01G6.4		Predicted E3 ubiquitin ligase	1.8	-	√	-	VA, DA
VF11C1L.1	*ppk-3*	PIP kinase	1.8	-	√	-	VA, DA
C25D7.8		Novel	1.9	-	√	-	VA, DA
F08G12.1			3.0	-	√	-	VA, DA
M79.1	*abl-1*	Abelson kinase	2.3	-	√	-	VA, DA
F25G6.4	*acr-15*	Acetylcholine receptor	-	4.9	√	-	VA, DA
T27A1.6	*mab-9*	Transcription factor	-	1.7	√	-	DA
F39G3.8	*tig-2*	TGF-β	-	1.8	√	-	VA, DA
T19C4.5		Novel	-	2.0		-	
CC4.2	*nlp-15*	Neuropeptide	-	6.5	√	-	
C18H9.7	*rpy-1*	Rapsyn	-	2.7	√	-	DA
Y71D11A.5		Ligand-gated ion channel	2.1	1.8	√	-	
C04E12.7		Phospholipid scramblase	-	3.2	√	1.8	VA, DA
F36A2.4	*twk-30*	K^+ ^channel	-	2.1	√	5.1	VA, DA
Y71H9A.3	*sto-4*	Stomatin	-	3.0	√	1.6	VA
F29G6.2		Novel	-	3.2	√	1.6	VA, DA,SAB, I5, AVF
C44B11.3	*mec-12*	Alpha-tubulin	-	5.9	√	1.9	VA, DA
T23D8.2	*tsp-7*	Tetraspanin	-	3.5	√	4.8	VA, DA
T05C12.2	*acr-14*	Acetylcholine receptor	-	1.5	√	3.1	DA
F33D4.3	*flp-13*	Neuropeptide	-	7.1	√	7.9	I5
C11D2.6	*nca-1*	Ca^++ ^channel	-	2.3	√	2.2	VA, DA
E03D2.2	*nlp-9*	Neuropeptide	-	3.1	√	2.5	VA
F55C12.4		Novel	-	3.5	√	2.1	DA
F43C9.4	*mig-13*	CUB domain	-	1.8	√	2.8	VA, DA
F39B2.8		Predicted membrane protein	1.7	3.5	√	2.1	VA, DA
K02E10.8	*syg-1*	Ig domain	1.8	1.8	√	1.8	VA, DA
ZC21.2	*trp-1*	Ca^++ ^channel	1.9	2.2	√	1.9	VA, DA
Y47D3B.2a	*nlp-21*	Neuropeptide	3.9	8.3	√	3.7	VA, DA
F09C3.2		Phosphatase	1.9	2.7	√	1.7	VA, DA
T27E9.9		Ligand-gated ion channel	2.3	4.0	√	3.1	
Y34D9B.1	*mig-1*	Frizzled-like	-	-	√	1.6	VA, DA

*GFP expression in neurons (check mark), and in A-class motor neurons (DA, VA, SAB, I5). GFP expression was typically determined in L2 larvae. Full expression patterns can be found in Additional data file 17. Expression patterns for some of these GFP reporters have been previously reported: T27A1.6, F39G3.8, T19C4.5, CC4.2, C18H9.7, F36A2.4, F29G6.2, T23D8.2, T05C12.2, F33D4.3, C11D2.6, E03D2.2, F55C12.4, F43C9.4, K02E10.8, ZC21.2, Y47D3B.2a, F09C3.2 [5]; F33D4.3 [43]; CC4.2, E03D2.2 [96]; F36A2.4, [121]; F43C9.4, [122]. Y47D3B.2a, [123].

### Gene families enriched in neurons of *C. elegans *larvae

Protein-encoding genes in the enriched larval pan-neural profile were organized into groups on the basis of KOGs and other descriptions that identify functional or structural categories (Table 2; Additional data file 4) [20]. Over half (880/1,562) are homologous to proteins in at least one other widely diverged eukaryotic species (that is, KOGs and TWOGs), 49 of which are classified as uncharacterized conserved proteins. Homologs for an additional 225 pan-neural enriched proteins are limited to other nematode species (that is, LSEs).

**Table 2:** Transcripts enriched in *C. elegans *neurons

Category	Embryonic pan-neural	Larval pan-neural	Embryonic A-class	Larval A-class
**Ion channels/receptors/membrane proteins**	122	156	60	41
Acetylcholine receptors	13	24	9	9
GABA receptors	1	4		3
Glutamate receptors	8	8	1	2
Potassium channels	11	24	8	10
Calcium channels	8	10	7	4
DEG/ENaC channels	3	10	1	1
Stomatins	3	7	2	1
Other ligand-gated ion channels	6	13	2	2
Gap junction proteins (innexins)	4	4	1	1
Symporters/exchangers/transporters	24	27	12	3
Other membrane proteins	41	25	17	5
				
**Axon guidance**	4	8	8	3
				
**Adhesion/Ig domain**	6	17	10	11
				
**Cytoskeleton-related**	33	34	16	5
				
**Transcriptional control**	90	91	38	10
Homeobox	8	28	3	3
Hormone receptors	24	15	5	1
Aryl-hydrocarbon receptors	1	3	1	
SMADs		3	1	1
HMG box	5	5		
HLH factors	2	4	1	1
Other transcription factors	32	25	13	4
General factors	18	8	14	
				
**Kinase/phosphatase**	82	79	51	18
				
**GPCR signaling**	107	169	42	25
G-protein coupled receptors	85	137	33	18
G-proteins	8	10	3	3
Regulators of G-protein signaling (GTPases, GEFs, GRKs)	7	8	4	2
Adenylate/guanylate cyclases	7	14	2	2
				
**Rab/Rho/Rac GTPase signaling**	17	7	7	2
				
**Neuropeptides**	39	58	11	13
FMRFamide-like (flp)	13	20	4	5
Neuropeptide-like (nlp)	13	18	3	4
Insulin-like	9	11	2	1
TGF-beta	1	3	1	1
Pro-protein convertases	3	6	1	3
				
**Calcium binding**	18	26	12	9
				
**Synaptic vesicle associated**	38	53	25	17
				
**RNA binding**	22	14	22	3
				
**Ubiquitin associated**	39	19	12	3
				
**Enzymes**	199	103	111	30
				
**Collagens**	2	1	5	24
				
**Other**	297	205	174	44
				
**Unnamed/uncharacterized**	159	127	161	56
				
**Unclassified**	363	395	230	98
				
**Total**	1,637	1,562	995	412

**Figure 7:**
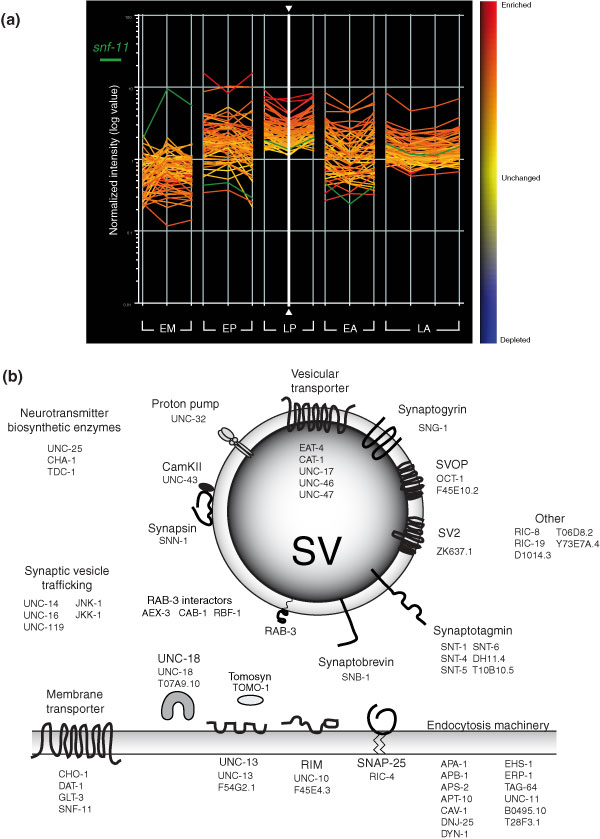
Transcripts encoding proteins that function in synaptic transmission are enriched in the neural datasets but largely excluded from muscle. **(a) **The line graph depicts 61 synaptic transmission genes that are enriched in the larval pan-neural (LP) dataset (colors from heat map at right are defined by LP sample denoted by vertical white line with arrowheads). Most of these transcripts are also enriched in other neuronal datasets (embryonic pan-neural (EP), embryonic A-class motor neuron (EA), larval A-class motor neuron (LA)) but not in embryonic muscle (EM). An exception is *snf-11 *(horizontal green line), the membrane-bound GABA transporter, which is significantly elevated in the EM and LP datasets, consistent with its known expression in muscle and neurons [26]. **(b) **Many of the proteins encoded by the 61 LP-enriched synaptic transmission genes are localized to synaptic vesicles (SV; center circle) or to the plasma membrane (shaded rectangle). Other proteins are predicted to perform related functions, such as the synthesis of neurotransmitters and/or vesicular trafficking.

Transcripts encoding proteins with fundamental roles in neuronal activity or signaling are highly represented in this dataset (for a comprehensive list see Additional data file 4). For example, in addition to the 34 synaptic vesicle (SV) associated transcripts from Figure 3b (Additional data file 7), transcripts for 19 proteins with potential roles in synaptic vesicle function are identified (Figure 7). These include six members of the synaptotagmin family of calcium-dependent phospholipid binding proteins (*snt-1*, *snt-4*, *snt-5*, *snt-6*, DH11.4, T10B10.5), only one of which, *snt-1*, has been previously shown to function in neurons [21]. Expression of the additional synaptotagmin genes in the nervous system may account for the residual synaptic vesicle function of *snt-1 *mutants [21]. Three members of the copine family (B0495.10, *tag-64*, T28F3.1), a related group of calciumbinding proteins with potential roles in synaptic vesicle fusion (listed as part of endocytosis machinery in Figure 7), are also enriched [22].

In addition to genes with general functions in synaptic vesicle signaling, the larval pan-neural profile includes transcripts encoding proteins with roles specific to particular neurotransmitters. For example, the plasma membrane and vesicular transporters for choline and acetylcholine (*cho-1 *and *unc-17*), GABA (*snf-11 *and *unc-46*, *unc-47*), dopamine (*dat-1 *and *cat-1*), and glutamate (*glt-3 *and *eat-4*) are included (Figure 7) [23-27]. The corresponding families of neurotransmitter-specific ligand-gated ion channels are highly represented, including 22 members of the ionotropic nicotinic acetylcholine (ACh) receptor family (Additional data file 4). Other classes of ion channels with key neural functions are also abundant, such as potassium channels (24), voltage-gated calcium channels (10) and DEG/ENaC sodium channels (10) (Table 2).

The wide range of neurotransmitter-specific genes in the larval pan-neural dataset reflects the diverse array of neuron types in *C. elegans *(Figure 5). This point is underscored by the detection of a large number of transcription factors with established roles in neuronal specification (Table 3). These include UNC-86, the POU homeodomain protein that regulates the differentiation of a broad cross-section of neuron classes [28-30], as well as transcription factors that define specific neuronal subtypes, such as the canonical LIM homeodomain MEC-3 (mechanosensory neurons) [31-33] and the UNC-4 homeodomain (A-class ventral cord motor neurons, see below) [34-37]. Transcription factors with undefined roles in the nervous system are also identified. Of particular note are 15 members of the nuclear hormone receptor (NHR) family, only one of which, *fax-1*, has been previously shown to regulate neuronal differentiation [38].

**Table 3:** Major transcription factor families enriched in *C. elegans *neurons

Transcription factor families		Fold change			
	
Cosmid name	Common name	Embryonic pan-neural	Larval pan-neural	Embryonic A-class	Larval A-class
**Homeobox**					
C40H5.5	*ttx-3*	1.6	1.5		
C33D12.1	*ceh-31*		1.6		
D1007.1	*ceh-17*		1.6		
K02B12.1	*ceh-6*		1.6		1.6
T13C5.4		3.3	1.6		
T26C11.7	*ceh-39*		1.6		
ZC64.3	*ceh-18*		1.6		
C18B12.3			1.7		
C28A5.4	*ceh-43*		1.7		
F56A12.1	*unc-39*		1.8		
W03A3.1	*ceh-10*		1.8		
C10G8.6	*ceh-34*		1.9		
C17H12.9			2		
F55B12.1	*ceh-24*		2		
ZC123.3		1.6	2		
C30A5.7	*unc-86*		2.1		
T26C11.5	*ceh-41*	1.6	2.1		
C39E6.4	*mls-2*		2.3		
F01D4.6	*mec-3*		2.6		
R08B4.2			2.6		
B0564.10	*unc-30*	2.2	2.7		
W05E10.3	*ceh-32*		2.7		
F26C11.2	*unc-4*		2.8	13.2	9.0
C37E2.4	*ceh-36*		2.9		
R07B1.1	*vab-15*		2.9		
Y54F10AM.4	*ceh-44*		3.3		2.1
F58E6.10	*unc-42*	2.4	3.8		
ZC247.3	*lin-11*		5.2		
C07E3.5		1.7			
F46C8.5	*ceh-14*	1.7			
W06A7.3	*ret-1*			1.8	
Y113G7A.6	*ttx-1*			2.8	
					
**Hormone receptors**					
Y94H6A.1			1.5		
F47C10.3			1.6		
F47C10.7			1.6		
F56E3.4	*fax-1*		1.7		
H01A20.1	*nhr-3*	1.8	1.7	1.9	
R09G11.2	*nhr-1*		1.7		
T03G6.2	*nhr-40*		1.7		
Y39B6A.17	*nhr-95*	1.6	1.7	1.8	
C24G6.4	*nhr-47*	2	1.8		
K06B4.8			2		
K06B4.1	*nhr-51*	2.5	2.1		
K06B4.2	*nhr-52*		2.1		
F21D12.1	*nhr-21*		2.2		
C49F5.4			2.9		
F07C3.10	*nhr-63*		4		
C06C6.4	*nhr-67*	1.8			
C08F8.8	*nhr-124*	1.5			
C17E7.8	*nhr-116*	2.3			
F09C6.9		3.3		1.9	
F16B4.9		1.9			
F31F4.12		1.6			
F41B5.9	*nhr-96*	2.5		1.7	
F44C8.11		1.8			
F44C8.9		1.7			
F48G7.11		2			
F59E11.8		1.8			
K06B4.10	*nhr-88*				1.8
K08A2.5	*nhr-71*	1.5			
K11E4.5		1.8			
R07B7.15	*nhr-104*	1.7			
R11E3.5				1.8	
T07C5.4	*nhr-44*	3.2			
T19A5.4	*nhr-59*	1.9			
T27B7.1	*nhr-115*	1.7			
T27B7.4	*nhr-65*	2.9			
Y17D7A.3		1.5			
Y67D8B.2		1.8			
					
**Aryl-hydrocarbon receptors**					
C25A1.11	*aha-1*		1.7		
C41G7.5	*ahr-1*		1.7		
C56C10.10		2.2	2	2.3	
					
**SMADs**					
F01G10.8	*daf-14*		1.7		
F25E2.5	*daf-3*		1.9		
F37D6.6	*tag-68*		2.2	2.3	2.0
					
**HMG box**					
F40E10.2	*sox-3*	1.6	1.5		
T22B7.1	*egl-13*		1.6		
T05A7.4	*hmg-11*	2.3	2.1		
F47G4.6			2.3		
K08A8.2	*sox-2*	2.5	2.9		
C12D12.5		4.7			
Y17G7A.1	*hmg-12*	1.8			
					
**HLH factors**					
C43H6.8	*hlh-15*	4.4	1.6		
F58A4.7	*hlh-11*		1.6		
Y16B4A.1	*unc-3*		2.9	3	4.9
F48D6.3	*hlh-13*		3		
W02C12.3		1.8			

A striking example of the power of this profiling approach is revealed by strong enrichment for genes involved in peptidergic signaling. Neuropeptides are potent modulators of synaptic transmission. A combination of genetic and pharmacological experiments have assigned specific neuromodulatory roles to FMRFamide and related peptides (FaRPs) encoded by members of the '*flp*' (FMRFamide like peptides) gene family [39]. Examples include *flp-13 *(cell excitability)[40], *flp-1 *(locomotion) [41] and *flp-21 *(feeding behavior) [42]. The enriched status of the majority of *flp *genes (20/23) in the larval pan-neural profile (Figure 4b) parallels immunostaining and GFP reporter results showing expression of this gene family in the *C. elegans *nervous system [43]. Transcripts encoding insulin-like peptides (*ins*) and neuropeptide-like genes (*nlp*) are among the most highly enriched mRNAs in the pan-neural dataset (Additional data file 4). Neuropeptide activating proteases such as the proprotein convertase *egl-3 *and the carboxypeptidase *egl-21 *are also elevated [44]. Finally, we detect 136 members of the G-protein coupled receptor (GPCR) family, including four GPCRs (*npr-1*, *npr-2*, *npr-3 *and T19F4.1) that have been either directly identified as neuropeptide receptors or implicated in neuropeptide-dependent behaviors [42,45,46] (E Siney, A Cook, N Kriek, L Holden Dye, personal communication). The strong representation of diverse neuropeptidergic components in the larval pan-neural profile is suggestive of a nervous system that is richly endowed with complex signaling pathways for modulating function and behavior.

### Embryonic and larval nervous systems express many common sets of genes

**Figure 8:**
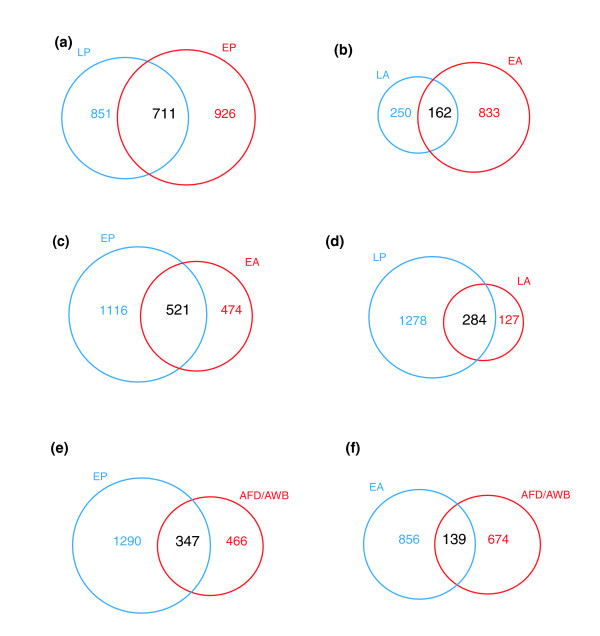
Venn diagrams comparing transcripts from profiled cell types at specific developmental stages. **(a) **Larval pan-neural (LP) and embryonic pan-neural (EP) datasets are enriched for common transcripts, but also contain transcripts exclusive to either developmental stage. **(b) **Larval A-class (LA) and embryonic A-class (EA) identify 162 shared transcripts. Transcripts selectively enriched in either neuron type may contribute to the unique morphologies of DA versus VA motor neurons (Figure 10). **(c,d) **The depth of the pan-neural datasets (EP, LP) is reflected in the substantial overlap with the A-class motor neuron profiles (EA, LA). Genes exclusively enriched in the EA and LA profiles are indicative of rare transcripts showing neuron specific expression. **(e,f) **Comparisions of the embryonic neural specific datasets (EP, EA) described in this paper with the embryonic profile of specific thermosensory neurons (AFD and AWB described by Colosimo *et al*. [8]. The AFD/AWB profile shows greater overlap with the EP dataset (e) than with the EA profile (f). See Additional data files 10 and 11 for lists of genes identified in each comparison.

To complement the profile of the larval nervous system obtained by the mRNAtagging method, a pan-neural GFP reporter gene [47] (J Culotti, personal communication) was used to mark embryonic neurons for MAPCeL analysis. GFP labeled neurons were isolated by FACS to ≥ 90% purity from primary cultures of embryonic cells (see Materials and methods). Comparisons of independent replicates showed that these data are highly reproducible (Additional data file 8). We identified 1,637 enriched genes (≥ 1.5-fold, FDR ≤ 1%) versus a reference dataset obtained from all embryonic cells (Additional data file 1). The majority (82%) of transcripts in this list with known expression patterns are expressed in neurons (Figure 3a). All of the promoter-GFP fusions (10/10) created from previously uncharacterized genes in the enriched embryonic pan-neural dataset showed expression in neurons, further validating this MAPCeL profile (Table 1; Additional data file 17). A comparison of the embryonic (MAPCeL) and larval (mRNA-tagging) profiles reveals considerable overlap, with approximately 45% of transcripts (710/1,637; representation factor 5.2, *p *< 1e^-325^) enriched in the embryonic neurons also elevated in larval neurons (Figure 8a). The intersection of these two datasets is significantly enriched (96%) for known neuron-expressed genes. The high likelihood of neural expression for these transcripts is underscored by our finding that a set of approximately 240 candidate neural genes originally identified as including a presumptive pan-neural regulatory motif ('N1 box') are overrepresented (35%, representation factor 2.6, *p *< 4.1e^-17^) in this subset of pan-neural transcripts [48].

As an additional test of the similarities between these independent datasets, we examined the embryonic and larval pan-neural profiles for elevated expression of gene families with roles in synaptic vesicle function (Figure 7a). Both the embryonic and larval pan-neural datasets were enriched for many of these components. In contrast, the majority of these transcripts are not upregulated in a MAPCeL profile of embryonic muscles (RMF, DMM, unpublished data). Interestingly, the one exception to this correlation, the GABA transporter *snf-11*, is known to be expressed in body wall muscle in addition to neurons [26].

**Figure 9:**
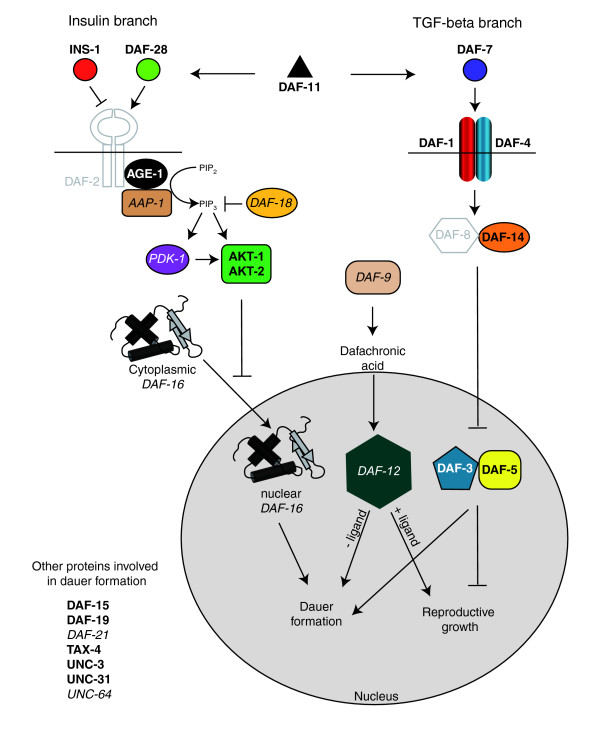
A majority of dauer pathway genes are enriched in either the larval pan-neural (LP) or embryonic pan-neural (EP) datasets. Two neuronal pathways influence the decision to dauer, an alternative developmental pathway adopted in unfavorable conditions [49-54]. During normal growth, the DAF-28 insulin-like molecule activates the DAF-2 insulin receptor to initiate a signal transduction pathway that prevents the translocation of the DAF-16 Forkhead transcription factor into the nucleus, thus blocking dauer formation. In a parallel pathway, DAF-7/TGF-beta activates receptors DAF-1 and DAF-4 to inhibit the Smad/Sno complex DAF-3/DAF-5, thereby promoting reproductive growth. The guanylyl cyclase DAF-11 drives expression of DAF-28 and DAF-7. During reproductive growth, the CYP2 cytochrome P450 enzyme DAF-9 is active and produces the DAF-12 ligand dafachronic acid. In the presence of its ligand, the nuclear hormone receptor DAF-12 promotes normal development. In the absence of its ligand, DAF-12 instead promotes dauer formation. Other proteins function independently of these pathways (for example, the DAF-19 transcription factor specifies ciliated neurons that detect exogenous dauer-inducing signals). Bold lettering denotes enriched transcripts and italics marks EGs detected in at least one of the pan-neural datasets. Gray letters refer to transcripts not found in either EP or LP datasets. See Additional data file 18 for a complete description of these genes.

Examination of the embryonic and larval pan-neural datasets confirmed expression of genes that regulate the dauer pathway in *C. elegans *neurons. The dauer larva adopts an alternative developmental program to withstand stressful conditions (for instance, starvation, overcrowding, high temperature). The decision to adopt the dauer state is regulated by the nervous system and is triggered during the L1/L2 transition in response to environmental cues [49-54]. Figure 9 graphically represents the dauer pathway genes identified in the combined pan-neural datasets. Of particular note is a conserved insulin-dependent signaling pathway (for example, *age-1*/PI3Kinase) that also regulates lifespan in *C. elegans *and in other species [28].

Transcription factors constitute the largest gene family that is differentially enriched between the embryonic and larval pan-neural profiles (Table 3). For example, the combined pan-neural datasets detect a total of 30 NHRs. However, 16 NHRs are exclusively detected in embryonic neurons, whereas only six are enriched solely in larval neurons. Homeodomain transcription factors are also unequally distributed across the two datasets. Of 32 enriched homeoproteins, 24 are exclusive to the larval pan-neural profile, whereas only 4 are selectively elevated in the embryonic pan-neural dataset (Table 3). The relative lack of enrichment of homeodomain mRNAs in the embryonic pan-neural profile was initially surprising given strong genetic evidence for the widespread role of the members of this transcription factor class in embryonic neural development [31,47,55-57]. A likely explanation for this finding is that many homeobox transcripts are dynamically expressed in multiple cell types in the embryo but are increasingly restricted to neurons during larval development [56,58]. This view is consistent with our observation that a majority (22/28) of homeodomain genes that are enriched in the larval pan-neural dataset are in fact also detected as expressed genes in the embryonic pan-neural profile (see below).

### Homologs of *C. elegans *neural genes are expressed in the mammalian brain

Over half of the enriched transcripts identified in the embryonic and larval pan-neural profiles have likely homologs in mammals (Additional data file 1). A substantial fraction of these transcripts encodes members of protein families with conserved roles in neural function or development (for instance, synaptic vesicle proteins; Figure 7b). We also identified neuron-enriched transcripts from *C. elegans *that are conserved but have largely undefined *in vivo *biochemical functions. For example, of the 711 transcripts that are enriched in both the embryonic and larval pan-neural datasets (Figure 8a), 27 encode uncharacterized conserved proteins (Additional data file 9). To determine if these transcripts are also detected in the mammalian brain, we queried the Allen Brain Atlas [59], which catalogs *in situ *hybridization results for 20,000 mouse transcripts (see Materials and methods). Of the 27 uncharacterized conserved genes from *C. elegans*, 26 have mouse homologs and 25 are included in the Allen Brain Atlas. We find that 76% (19/25) of these genes are detected in the mouse brain and, therefore, suggest that neural functions for these genes are likely conserved from nematodes to mammals. For instance, one member of this group of genes, *osm-12*, is the *C. elegans *homolog of a human disease gene, *BBS7*. Bardet-Biedle syndrome (BBS; OMIM 209900) is a rare, pleiotropic disorder with multiple pathologies (obesity, rod-cone dystrophy, cognitive impairment) [60]. At least 12 genes (*BBS1-12*) have been linked to this disease [61]. *osm-12 *and other BBS genes are highly expressed in ciliated neurons in *C. elegans *and genetic studies suggest key roles in intraflagellar transport [62]. These findings and additional work in other systems have led to the hypothesis that basal body dysfunction could be the root cause of BBS [63-66]. Thus, we propose that genetic studies in *C. elegans *of other uncharacterized conserved genes detected in the pan-neural enriched profile may be instructive.

### The *C. elegans *interactome identifies neuronal genes potentially involved in synaptic function

**Figure 10:**
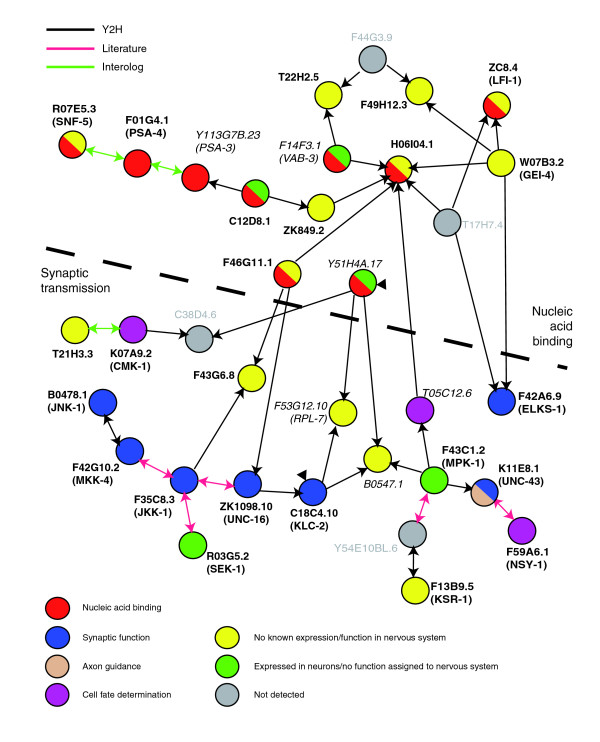
Interactome map of pan-neural genes. The *C. elegans *interactome contains functional and physical interactions for over 5,000 proteins. A comparison to the 711 transcripts enriched in both embryonic pan-neural (EP) and larval pan-neural (LP) datasets (see Figure 8) revealed a single large interaction cluster (see Materials and methods). Bold lettering denotes enriched transcripts and italics marks EGs detected in at least one of the pan-neural datasets. Gray letters refer to transcripts not found in either dataset. Black lines represent interactions isolated by yeast two-hybrid assay, red lines depict known interactions listed in worm Proteome Database (literature), and green lines denote *in silico *searches against orthologous pairs (interolog). Black arrows point from bait to prey. Arrowheads indicate self-interactions. Protein functions are denoted with colored circles (see key at bottom). The dashed black line demarcates two subgroups of interacting proteins, nucleic acid binding (above) versus synaptic transmission (below). See text for additional information.

The *C. elegans *interactome documents approximately 5,500 protein-protein interactions derived from yeast two-hybrid results, from interologs (that is, interactions between protein homologs in other species) and from functional interactions described in the literature [67]. To gain insight into the functional significance of prospective neural genes identified by these microarray datasets, we looked for evidence of interactions among proteins encoded by these genes in the Interactome database (see Materials and methods). The 711 transcripts enriched in both the embryonic and larval pan-neural datasets were uploaded for this analysis (Figure 8a). This search generated an interaction map with a single prominent cluster. Most of the transcripts in this group (30/34) are detected in at least one of the pan-neural datasets (Figure 10). Our finding that the majority of genes in this interactome group are expressed in the nervous system favors the idea that these networks reflect authentic interactions in neurons. We note that 13 of the proteins in this list (yellow circles in Figure 10) have not been previously assigned to the nervous system. Annotation of this interactome map with functional data for each corresponding protein revealed two distinct subclusters featuring roles in either synaptic transmission or nucleic acid binding. For example, the JIP3/JSAP1 JNK scaffolding protein, UNC-16, interacts with KLC-2 (kinesin light chain) to regulate vesicular transport in neurons [68]. Other members of this interacting complex, MKK-4 (MAP kinase kinase) and JNK-1 (Jun kinase) are also required for maintaining normal synaptic structure [69,70]. These findings suggest that additional proteins in this subcluster may function at the synapse. F43G6.8 (E3 ubiquitin ligase) and B0547.1 (COP-9 signalosome subunit) are attractive possibilities as synaptic development and function are regulated by ubiquitin-dependent protein degradation [71]. As more phenotypic data are compiled, this analysis can be extended to encompass data derived from RNA interference (RNAi) experiments, which may yield models for molecular machines that function in neurons [72].

### An mRNA-tagging transcriptional profile of a small subset of neurons

**Figure 11:**
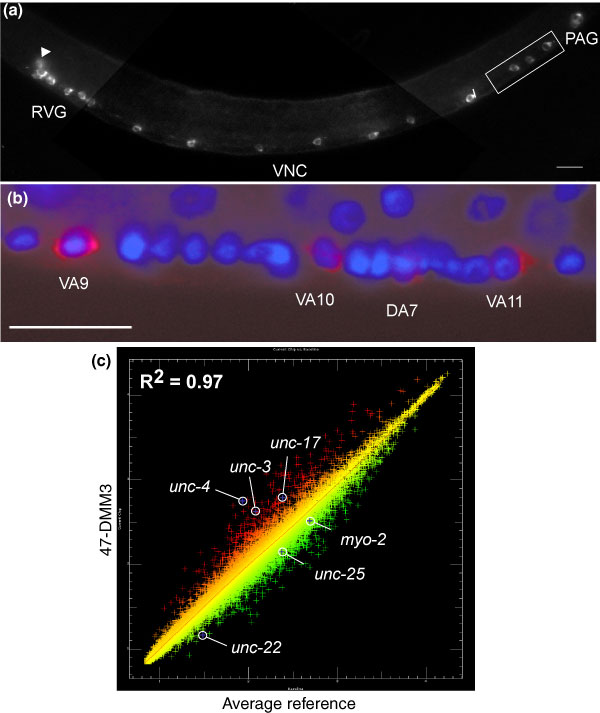
Larval A-motor neuron enriched transcripts are revealed by mRNAtagging with *unc-4*::3XFLAG::PAB-1. **(a) **Antibody staining detects FLAG::PAB-1 expression in A-class neurons in the retrovesicular ganglion (RVG), ventral nerve cord (VNC), and pre-anal ganglion (PAG) and in the I5 pharyngeal neuron (arrowhead). Lateral view of L2 larva, anterior is to left, ventral down. **(b) **Close-up of posterior ventral nerve cord (boxed area in (a)) showing that anti-FLAG staining (red) is restricted to cytoplasm of A-class motor neurons. DAPI (blue) marks cell nuclei (compare to Figure 1, in which all motor neurons show anti-FLAG staining). Anterior is left, ventral is down. Scale bars = 10 μm. **(c) **Results of a single larval A-class hybridization (47-DMM3; red) compared to average reference intensities (green) to identify differentially expressed transcripts. The known A-class genes *unc-4 *(homeodomain, A-class neurons), *unc-3 *(O/E transcription factor, cholinergic VNC motor neurons), and *unc-17 *(VAChT, cholinergic neurons) are enriched (red) in 47-DMM3. Genes expressed in other classes of neurons (*unc-25*, GAD, GABAergic neurons) or other tissues (*myo-2*, pharyngeal muscle myosin; *unc-22*, body wall muscle structural protein) are depleted (green) relative to the reference profile.

Although our gene expression profiles of the embryonic and larval nervous systems provide a comprehensive list of transcripts that function in neurons, these data lack the spatial resolution to identify the specific neurons in which these transcripts are expressed. For instance, the dopamine transporter, *dat-1*, is highly enriched (15.9-fold) in the larval pan-neural dataset, but *dat-1 *expression is limited to eight dopaminergic neurons [73]. Other transcripts that are also restricted to a small number of neurons, however, might not be detected in a global profile of the entire nervous system. For example, the genes *gcy-5 *and *gcy-6 *(guanylate cyclase) are each expressed in single neurons, ASER and ASEL [74], respectively, and neither is enriched in the larval pan-neural dataset. The application of the mRNA-tagging strategy to individual classes of neurons should, therefore, correlate gene expression with specific neurons as well as detect low abundance transcripts with potential key functions in these cells. To test this idea, we used the *unc-4 *promoter to express FLAG-PAB-1 in only the subset of neurons in the ventral nerve cord that express the UNC-4 homeodomain protein. In the L2 larva, *unc-4*::GFP and *unc-4*::LacZ reporters show strong expression in a total of 18 neurons: VA motor neurons (12), SAB motor neurons (3), the I5 pharyngeal motor neuron (1) and AVF interneurons (2) [35,75]. Weaker, sporadic expression is observed in nine embryonically derived DA motor neurons at this stage. (*unc-4 *is strongly expressed in the DAs in the embryo and in L1 larvae.) To increase the sensitivity of the mRNA-tagging method for profiling these neurons, PAB-1 was labeled with three tandem repeats of the FLAG epitope (3XFLAG). Figure 11a,b show a mid-L2 larval animal (NC694) expressing the *unc4*::3XFLAG::PAB-1 transgene in VA, SAB, and I5 motor neurons and in AVF interneurons; less intense expression is seen in the DA motor neurons. Because most (24/27) of the neurons in this group are members of the 'A-class' of ventral cord excitatory motor neurons (VA, SAB, DA), we will refer to the mRNA-tagging data obtained from this transgene as the 'larval A-class motor neuron' profile (Figure 9).

As previously observed for the larval pan-neural data (Figure 2), independent hybridizations resulted in highly reproducible data for the larval A-class motor neuron profile (Additional data file 8). A comparison of the A-class hybridization data to the reference sample of mRNA from the average larval cell detected 412 enriched genes (see Materials and methods). Of the 114 genes in this list with known expression patterns, 102 (approximately 90%) are found in neurons (Figure 3a). Of these genes, 96 have detailed spatial information, and 76 (approximately 80%) of these show annotated expression in regions that also contain UNC4expressing neurons (Additional data file 1). Of particular note, the native *unc-4 *transcript, which is selectively expressed in these neurons *in vivo*, is the most highly enriched (eight-fold) mRNA in this dataset. Other known A-class motor neuron genes in this list include the vesicular ACh transporter (VAChT) *unc-17 *and the Olf/EBF transcription factor *unc-3 *(Figure 11c) [75,76]. In contrast, transcripts known to be restricted to other cell types, such as muscle (*myo-2*, *unc-22*) or GABAergic neurons (*unc-25*), are depleted from the A-class neuronal profile (Figures 4a and 11c). For instance, <2% of transcripts selectively expressed in larval germ line, intestine, or muscle (30/1926) are enriched in the larval A-class motor neuron profile (Additional data file 5) [13].

All of the GFP reporter lines (19/19) constructed for A-class enriched transcripts (Table 1; Additional data file 17) are expressed in UNC-4 neurons. For example, in the mid-L2 stage ventral nerve cord, *mec-12*::GFP is expressed in DA, VA, VB and VD motor neurons (Figure 6a,e) and *syg1*::GFP (Ig domain) is detected in DA and VA motor neurons among others (Figure 6g). These results strongly suggest that most of the genes in the UNC-4 neuron enriched dataset are expressed in these cells *in vivo*. Thus, these data indicate that the mRNA-tagging method can produce a reliable profile of subsets of neurons in *C. elegans*.

### A subset of pan-neural genes are expressed in larval A-class motor neurons

Nearly 70% of the larval A-class enriched transcripts (282/412) are also elevated in the larval pan-neural dataset (representation factor 8.2, *p *< 2.9e^-209^; Additional data file 10). As expected, genes with known functions in all neurons are highly represented in this group (Table 2). Synaptic vesicle associated transcripts that are widely expressed in the nervous system, such as *rab-3 *(G-protein), *snt-1 *(synaptotagmin) and *snb-1 *(synaptobrevin), are enriched in both datasets. Absences from the larval A-class profile are correlated with class-specific functions in neurons. For example, the 60 transcripts encoding proteins involved in synaptic transmission enriched in the larval pan-neural dataset include vesicular transporters for GABA (*unc-47*), glutamate (*glt-3*), dopamine/serotonin (*cat-1*) and acetylcholine (*unc-17*) (Figure 7b) [24]. The selective enrichment of the vesicular ACh transporter *unc-17 *in the larval A-class profile is consistent with the known cholinergic signaling capacity of A-class motor neurons [75]. In another striking example of neuron-specific gene expression, the '*mec*' genes, which are required for normal differentiation or function of mechanosensory neurons, are highly represented in the larval pan-neural dataset but are not detected in the larval A-class profile (Table 4) [77]. The one exception is the alpha-tubulin encoding gene, *mec-12*, for which enriched expression in A-class neurons was confirmed with a GFP reporter gene (Figure 6a,e). As described above, most of the known *flp *genes are enriched in the pan-neural dataset [39]. A subset of five *flp *genes is found in the A-class dataset (*flp-2*, *4*, *5*, *12*, *13*), providing enhanced spatial resolution for the expression repertoire of this large family of neuropeptide transmitters (Figure 4b).

**Table 4:** Genes expressed in mechanosensory neurons are differentially detected in the larval pan-neural dataset versus the larval A-class dataset

Cosmid name	Gene name	Fold change		Description
			
		Pan-neural	A-class	
F14D12.4	*mec-2*	2.9	-	Prohibitins and stomatins of the PID superfamily
F01D4.6	*mec-3*	2.6	-	Transcription factor, contains HOX domain
T01C8.7	*mec-4*	2.7	-	Non voltage-gated ion channels (DEG/ENaC family)
W02D3.3	*mec-6*	1.9	-	Unnamed protein
ZK154.3	*mec-7*	3.6	-	Beta-tubulin
F16F9.5	*mec-10*	1.8	-	Non-voltage-gated ion channels (DEG/ENaC family)
F57H12.7	*mec-17*	7.6	-	Uncharacterized conserved protein
C52B9.9	*mec-18*	3.1	-	Acyl-CoA synthetase
C44B11.3	*mec-12*	5.9	1.9	Alpha-tubulin

**Figure 12:**
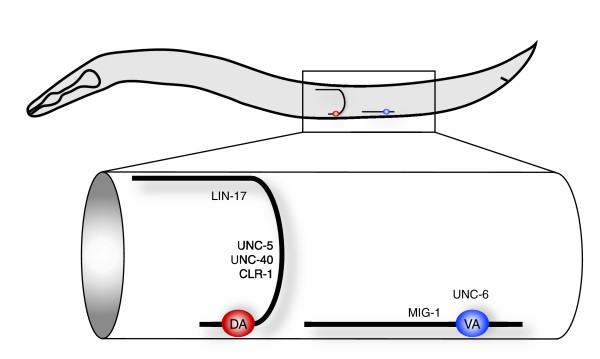
Differential expression of axon guidance cues and receptors in A-class motor neurons. Embryonic DA motor neurons extend commissures to innervate muscles on the dorsal side whereas larval VA motor axons are retained in the ventral nerve cord. DA axons are steered dorsally by interaction of the repulsive cue UNC-6 (netrin) with receptors, UNC-40 (DCC) and UNC-5, and the CLR-1 receptor tyrosine phosphatase [90,91]. VAs do not express these receptors and project ventrally directed axons. Enrichment of the Wnt receptors *lin-17 *in the embryonic A-class motor neuron dataset and *mig-1 *in the larval A-class motor neuron profile could be indicative of a Wnt-dependent mechanism for directing anterior outgrowth of DA and VA motor axons.

The A-class profile includes approximately 130 transcripts that are not detected in the larval pan-neural dataset (Additional data file 10). Interestingly, approximately 20% of these genes (23/127) encode collagen-like proteins for which neural functions are largely undefined. *cle-1*, which encodes a type XVIII collagen, the one member of this protein family that does have a documented role in the nervous system [78], is enriched in both the larval pan-neural and A-class datasets. We speculate that post-embryonic motor neurons may secrete collagens and other extracellular matrix components for assembly into the basement membrane that envelopes the ventral nerve cord [79]. Indeed, our data confirm that UNC-6 (netrin), a critical extracellular matrix signal that steers migrating cells and neuronal growth cones, is highly expressed in larval A-class motor neurons (Figure 12) [80].

### Comparison of transcripts enriched in embryonic versus larval A-class motor neurons

We have previously used the MAPCeL strategy to profile embryonic motor neurons marked with *unc-4*::GFP [5]. These include 12 embryonic A-class motor neurons (9 DA and 3 SAB) and a single pharyngeal neuron, I5 [5]. The embryonic A-class motor neurons are similar to the post-embryonic VAs in that they express *unc-4*, are cholinergic, extend anteriorly directed axons, and receive inputs from the command interneurons AVA, AVD, and AVE [79]. The strong overlap of these distinct morphological and functional traits as well as some residual larval expression of *unc-4 *in embryonic A-class motor neurons (Figure 11b) are consistent with the observation that approximately 40% of transcripts enriched in the larval A-class motor neuron dataset (162/412) are also elevated in the embryonic A-class motor neuron MAPCeL profile (representation factor 7.4, *p *< 3.1e^-99^; Figure 8b; Additional data file 10). Transcripts from the cholinergic locus, *cha-1 *(choline acetyl transferase) and *unc-17 *(vesicular ACh transporter), which are essential for the biosynthesis and packaging of ACh into synaptic vesicles, are enriched in both A-class motor neuron profiles [24]. In addition to these gene families, several others are enriched in both embryonic and larval A-class motor neurons (Additional data file 19). ACh signaling depends on the synaptic vesicle cycle and genes with key roles in this mechanism are elevated in both datasets: these include *unc-18*, *snt-1 *(syntaxin), *snn-1 *(synapsin), *ric-4 *(SNAP-25), *sng-1 *(synaptogyrin), *unc-2 *(calcium channel), *rab-3*, and *unc-11 *(clathrin component). In addition, genes with either established or likely roles in the G-protein coupled signaling pathways that modulate ACh release from these motor neurons (*dop-1*, *pkc-1*, *kin-2*, *gar2*, *rgs-1*, *rgs-6*, *gpc-2*) are common to both enriched datasets [5,81]. The general role of A-class motor neurons in both releasing and responding to a broad range of neuroactive signals is underscored by the embryonic and larval enrichment of multiple neuropeptides (that is, *flp-2*, *flp-4*, *flp-5*, and *flp-13*) (Figure 4B). Shared ionotropic receptors include the nAChR subunits, *acr-12*, *acr-14 *and *unc-38*, which lead to excitatory responses, as well as the recently described ACh gated chloride subunit, *acc-4 *(T27E9.9), which should mediate acetylcholine-induced inhibition of motor neuron activity [82]. Together, these data support the proposal that *C. elegans *A-class motor neurons utilize complex mechanisms for integrating signals originating as either paracrine or autocrine stimuli [5].

Other transcripts that are highly enriched in both embryonic and larval A-class datasets with potential roles in specifying shared characteristics of this motor neuron class include: *syg-1*, which encodes an Ig-domain membrane protein that localizes the presynaptic apparatus of the HSN motor neuron in the egg laying circuit (Figure 6g) [83]; *rig-6*, which encodes the nematode homolog of contactin, a membrane protein with extracellular fibronectin and Ig domains that organizes ion channel assemblages [84,85]; and *cdh-11*, which encodes the homolog of calsyntenin, a novel cadherin-like molecule that is highly localized to postsynaptic sites [86]. Finally, we note that of the 25 genes that encode innexin gap junction components [87], only one, *unc-9*, is enriched in both of the A-class motor neuron datasets. This finding points to the UNC-9 protein as a likely component of gap junctions that couple A-class motor neurons with command interneurons that drive motor circuit activity in the ventral nerve cord [37].

In addition to genes that are enriched in both embryonic and larval A-class motor neurons, we also detected transcripts that are selectively elevated in one or the other dataset (Additional data file 10). Transcription factors comprise the largest group of differentially expressed genes. Of 24 transcription factor genes enriched in embryonic A-class motor neurons, only two, *unc-3 *and *unc-4*, are also included in the separate list of 10 transcription factors enriched in larval A-class motor neurons (Table 3). UNC-3 (O/E HLH protein) and UNC-4 (homeodomain protein) have been previously shown to specify shared characteristics of embryonic and larval A-class motor neurons [36,75,76]. Roles for the remaining transcription factors in the differentiation of these motor neuron subtypes are unknown. For example, members of the POU (*ceh-6*) and CUT (*ceh-44*) classes of homeodomain protein families, which are well-established determinants of neuronal fate [88,89], are selectively enriched in the larval A-class list. Conversely, five members of the nuclear hormone receptor family (*nhr-3*, *nhr-95*, *nhr-104*, *nhr-116 *and F41B5.9) are preferentially expressed in embryonic A-type motor neurons. The extent to which these different combinations of transcription factors account for characteristics that distinguish embryonic and larval A-class motor neurons can now be explored by genetic analysis.

A key morphological feature that distinguishes DA from VA motor neurons is clearly linked to differential levels of specific transcripts in embryonic versus larval A-class datasets. During embryonic development, DA motor neurons extend commissures that circumnavigate the body wall to innervate dorsal muscles. The dorsal trajectory of DA motor neuron outgrowth depends on the UNC-6/netrin receptor genes, *unc-5 *and *unc-40*, and the receptor protein tyrosine phosphatase (RPTP) *clr-1 *gene [90,91], all three of which are enriched in the embryonic A-class dataset (Figure 12). In contrast, *unc-5*, *unc-40 *and *clr-1 *are not elevated in larval VA motor neurons, which consequently innervate muscles on the ventral side. Guidance cues that govern the anteriorly directed outgrowth of motor axons, the dorsal and ventral nerve cords, respectively, are not known. However, a likely candidate to direct axonal outgrowth along the *C. elegans *anterior-posterior axis is Wingless (Wnt) signaling [92-94]. In this regard, it is interesting that a comparison of the embryonic and larval A-class motor neuron transcripts identifies two different Wnt receptors that are selectively enriched in either the DA (*lin-17*) or VA (*mig-1*) motor neurons. In addition, the transcript for the Wnt ligand *cwn-1 *shows elevated expression in the embryonic A-class dataset.

### Comparisons to microarray profiles of *C. elegans *sensory neurons identify differentially expressed transcripts

Colosimo *et al*. [8] used MAPCeL to profile the sensory neurons AFD and AWB. We found that <20% of AFD/AWB enriched transcripts also show elevated expression in embryonic A-type motor neurons (Figure 8f; Additional data file 11), a finding consistent with the distinct roles of these neuron classes in *C. elegans*. For example, the AFD-specific guanylate cyclase genes, *gcy-8 *and *gcy-23*, are excluded from the enriched embryonic A-type motor neuron dataset, whereas the A-class specific transcription factor, *unc-4*, is not found in the AFD/AWB profile (Additional data file 11). In contrast, a significantly larger fraction (approximately 43%) of AFD/AWB enriched transcripts, including *gcy-8 *and *gcy-23*, are elevated in the embryonic pan-neural profile (Figure 8e) (Additional data file 11). Similar results were obtained when comparing the larval pan-neural and A-class datasets to a larval profile of chemosensory neurons [14] (data not shown). These findings confirm the reliability of these neuron-specific profiling methods for identifying differentially expressed transcripts and confirm that the panneural profiling approach is sufficiently sensitive to detect genes expressed in diverse cell types throughout the *C. elegans *nervous system.

### Microarray profiles are consistent with gene expression topographic maps

We compared our data to a topographic map derived from 553 microarray experiments in which genes are assigned to specific 'mountains' based on similarities in gene expression [95]. In some instances, co-regulated genes were grouped into specific functional subsets, thereby defining the 'name' of the mountain. For example, mountain 6 contains many genes that are known to function in neurons. Neuronal transcripts identified in all four of our neuronal microarray experiments (embryonic and larval pan-neural, embryonic and larval A-class) are significantly over-represented in the neuromuscular mountain (mountain 1) and one of the neuronal mountains (mountain 6). In contrast, transcripts in the embryonic muscle dataset are significantly under-represented in mountains 1 and 6 but are over-represented in the muscle mountain (mountain 16) (RMF, DMM unpublished data). These data provide additional validation for our neuronal expression profiles.

### Detection of expressed genes

We limited the analysis above to transcripts that show a statistically significant level of enrichment in neurons relative to other cell types in order to focus on genes that may function predominantly in the nervous system. Our microarray data, however, also include intensity values for a larger group of transcripts that may be broadly expressed in neurons as well as in other tissues. We define these transcripts as 'expressed genes' (EGs). We identified 7,953 EGs in the MAPCeL profile of embryonic neurons using criteria that exclude transcripts that are likely to originate from the small fraction (approximately 10%) of non-GFP cells in the FACS preparation [5] (Additional data file 12). For the larval pan-neural and larval A-class motor neuron datasets obtained with the mRNA-tagging method, EGs were defined using similar considerations, in this case, to exclude transcripts that are likely due to background levels of RNA adhering nonspecifically to the sepharose beads used in the immunoprecipitation step (see Materials and methods). EGs in these experimental samples represent transcripts that may be enriched in neurons as well as genes that are expressed at comparable levels in neurons and in other tissues. This approach identified a total of 4,033 EGs in the larval pan-neural dataset and 3,320 EGs in the larval A-class profile (Additional data file 13). As expected, 'housekeeping' genes are prevalent in these datasets but excluded from the neuron enriched profiles. For example, 20 ribosomal subunit genes (13 large, 7 small) are included in the dataset of larval pan-neural EGs but are not listed in the profile of transcripts enriched in larval neurons (Additional data files 1 and 13).

A comparison of all EGs in the larval and embryonic datasets described in this paper (that is, reference, pan-neural, A-class motor neurons), in addition to the previously described embryonic A-class dataset [5], reveals a total of approximately 12,000 unique transcripts or 63% of the predicted genes represented on the *C. elegans *Affymetrix Gene Chip (Additional data file 14). We note that approximately 1,600 of these EGs correspond to transcripts that have not been previously confirmed by expressed sequence tags (Additional data file 16); a subset of 336 transcripts from this group is enriched in at least one of the neuronal datasets, suggesting that they may have specific functions in *C. elegans *neurons.

## Discussion

We have used two complementary microarray-based strategies to obtain comprehensive gene expression profiles of developing *C. elegans *neurons. In the MAPCeL method, GFP-labeled embryonic neurons were isolated by FACS for microarray profiling [5]. Because postembryonic neurons are not readily available for sorting [12], we used an alternative strategy, the mRNA-tagging method, to profile the larval nervous system [11]. In this approach, neuronal mRNAs were purified by immunoprecipitation from transgenic animals expressing an epitope-tagged RNA binding protein (FLAG-PAB-1) in larval neurons. Together, these microarray datasets identify 2,488 transcripts that show elevated expression in the *C. elegans *nervous system relative to other tissues in at least one developmental stage (that is, embryonic or larval) (Additional data file 10). A bioinformatic query of WormBase confirmed enrichment of known neural transcripts in these datasets (Figure 3a). In addition, analysis of a representative group of newly constructed GFP reporters has confirmed *in vivo *neural expression of >90% of previously uncharacterized genes on these lists (Table 1). We therefore conclude that these 'panneural' profiles provide accurate representations of gene expression in the *C. elegans *embryonic and larval nervous systems. These transcripts encode proteins with a broad array of functions. For example, as expected, ion channels, neurotransmitter receptors and synaptic vesicle components are highly represented (Figure 7; Table 2; Additional data file 4). In a striking indication of the complex signaling capacity of the *C. elegans *nervous system, most of the known peptide neurotransmitter genes (for example, 20 of 23 FMRFamide genes or '*flps*') are enriched in the larval pan-neural dataset (Figure 4; Additional data file 4) [96]. Neural functions for previously uncharacterized members of these gene families can now be assigned by genetic or RNAi analysis. With this possibility in mind, we tested the applicability of these expression data for predicting *in vivo *functions for genes in this dataset that are also included in a genome-wide interaction map or 'interactome' for *C. elegans *proteins [67]. This analysis revealed that proteins encoded by a subset of panneural transcripts are linked to identified components of the synaptic vesicle cycle and, therefore, predicts that genetic or RNAi perturbation of these genes should result in neurotransmitter signaling defects (Figure 10). In addition to finding transcripts that may have shared roles in both the embryonic and larval nervous system, these pan-neural profiles have also identified a significant number of genes (71%, 1,777/2,488) that are differentially enriched in either embryonic or larval neurons. In the future, it will be interesting to determine if these genes define stage-specific features of the developing nervous system.

### The mRNA-tagging method can be used to generate gene expression profiles of specific neurons

In addition to detecting transcripts that are broadly expressed throughout the nervous system (that is, synaptic vesicle components), the pan-neural profiles also include genes that are selectively expressed in specific neurons. In most instances, these known assignments are based on promoter-GFP reporter constructs for a limited number of genes in a given neuron and are, therefore, incomplete. To test the applicability of the mRNA-tagging strategy for obtaining a comprehensive gene expression profile of a specific subset of neurons, we utilized this approach to fingerprint a group of 18 larval cells largely composed of A-type motor neurons [35,75]. This experiment revealed >400 transcripts with enriched expression in these cells (Additional data file 1). Although the majority (70%) of these transcripts also show elevated expression in the larval pan-neural profile (Figure 8), a significant fraction of these mRNAs are exclusively enriched in the A-class dataset in this comparison and are, therefore, likely to represent genes with limited expression in the nervous system. These results indicate that the mRNA-tagging strategy can now be applied to monitor gene expression in specific *C. elegans *neurons and that this approach should detect neuron-specific genes with potential key roles in the specification or function of individual neuron types. Our findings confirm an earlier study in which a neuron specific promoter was used in conjunction with the mRNA-tagging strategy to identify transcripts that are highly expressed in a group of approximately 50 sensory neurons from *C. elegans *[14]. Our work provides the important technical advance, however, of substantially enhancing the sensitivity of this method; we show that reliable profiles can be obtained by amplifying nanogram quantities of mRNA whereas the method of Kunitomo *et al*. [14] required micrograms of starting mRNA.

### Limitations of the mRNA tagging method

Despite the successful use of mRNA-tagging for these cell-specific profiling experiments, additional improvements in this method would be helpful. For example, with any given promoter, we sometimes observe FLAG-1::PAB-1 staining in the expected cell types as well as in additional ectopic locations (data not shown). This problem is unlikely to result from gene expression domains in the transgenic PAB-1 construct because the substitution of *pab-1 *cDNA to remove all possible genomic PAB-1 regulatory sites did not rectify this problem (Von Stetina *et al*., unpublished data). Our solution has been to generate multiple transgenic lines for each construct until we obtain at least one line in which FLAG-PAB-1 expression is limited to the cells of choice. A second problem with this method is pull-down of non-specific mRNA bound to the anti-FLAG sepharose beads. We have reduced this background by including a stringent wash step with a low salt buffer, but additional treatments to remove this extraneous mRNA would enhance the sensitivity of this method (see Materials and methods). Lastly, some promoters result in subviable transgenic lines or unpredictable genetic interactions that limit profiling experiments [37] (data not shown). The biological mechanisms of these effects are unknown but have also been observed for PAB-1 mRNA-tagging lines in *Drosophila *[97].

### Applications of cell-specific microarray profiling methods

The mRNA-tagging strategy has been used to generate robust gene expression profiles of major *C. elegans *tissues (that is, muscles, intestine, nervous system) [11,13] (this paper). By exploiting promoter elements with more limited expression, it has also been possible to extend this approach to specific subsets of neurons. These results suggest that mRNAtagging can now be exploited to obtain gene expression profiles in a broad array of cell types at precisely defined developmental intervals. For example, mRNA-tagging profiles obtained during a critical larval period in which GABAergic motor neurons switch axonal versus dendritic polarity could potentially reveal genes that direct the remodeling process [98]. The combined profiling results reported in this paper identify a set of 177 transcription factors showing enriched expression in neurons. Genetic analysis has established that many of these transcription factors regulate key aspects of neuronal differentiation and function [31,47,55-57,76,99,100]. Both the MAPCeL and mRNA-tagging approaches can now be utilized to generate comparisons of mutant versus wild-type profiles that should reveal transcription factor-regulated genes in specific neurons [9,37]. Microarray profiling of mutants for other classes of proteins could also be utilized to reveal unexpected gene regulatory roles. For example, a comparison of pan-neural mRNA-tagging datasets obtained from mutant versus wild-type animals indicates that the conserved synaptic protein RPM-1/Highwire regulates gene expression throughout *C. elegans *nervous system (JDW, SEV, DMM, unpublished results). The *C. elegans *nervous system is uniquely well-defined with a wiring diagram denoting chemical synapses and gap junctions among all 302 neurons. It should now be possible to exploit these cell-specific microarray profiling methods to define genes expressed in each type of neuron in this circuit. In turn, novel computational methods could be exploited to link specific subsets of these genes to roles in defining the connectivity architecture of this network [101,102].

### Towards defining the transcriptome

In addition to transcripts showing elevated expression in neurons, our neural microarray profiles include a larger group of transcripts that are expressed in neurons and in other tissues at comparable levels. We refer to these transcripts as 'expressed genes'. A comparison of the three larval datasets described in this work (reference, larval pan-neural, larval A-class motor neuron) reveals that 1,424 EGs are shared and are, therefore, likely to represent transcripts that function in a broad array of cell types. In contrast, a smaller number of transcripts are uniquely detected in either the larval pan-neural (1,189) or larval A-class motor neuron (435) datasets. The three embryonic datasets (reference, embryonic pan-neural, embryonic A-class motor neuron) commonly express 4,995 EGs, with 280 EGs unique to embryonic A-class motor neurons and 480 mRNAs selectively detected in the embryonic pan-neural profile. These findings suggest that microarray-based strategies to confirm *in vivo *expression of all predicted *C. elegans *genes or to identify new, previously unknown transcripts (for example, tiling array profiles) [103], will require extraction of mRNA from a variety of specific cells and tissues with methods similar to those described here.

## Conclusion

Approximately 9,000 *C. elegans *genes represented on the Affymetrix array have annotated human homologs (Additional data file 3). Roughly 5% (525) of these genes encode uncharacterized conserved proteins. Our combined microarray data have revealed that 108 of these transcripts are enriched in neurons (Additional data file 24). The high conservation of this subset of genes from nematodes to humans indicates that the encoded proteins may play pivotal roles in neuronal function or specification. Indeed, we show that approximately 80% of the members of a core group of pan-neural genes (19/25) from this list are expressed in the mammalian brain. The MAPCeL and mRNA-tagging strategies provide sufficient temporal information to pinpoint the developmental period during which a gene may function, as well as the spatial resolution to define the neuron in which it is expressed. With the powerful molecular and genetic tools available to *C. elegans *researchers, it should now be possible to delineate the roles of these novel targets in the nervous system.

## Materials and methods

### Nematode strains

Nematodes were grown as described [104]. Strains were maintained on nematode growth media plates inoculated with the *E*. *coli *strain OP50 [105]. Strains used to isolate transcripts via mRNA-tagging were N2 (wild type), SD1241 (*gaIs153*, *F25B3.3*::FLAG::PAB-1) (NC694 (*wdEx257*, *unc-4*::3XFLAG::PAB-1) [37]. GFPtagged embryonic neurons were isolated from NW1229 (*evIs111*, *F25B3.3*::GFP) [47] (J Culotti, personal communication) for MAPCeL analysis.

### Molecular biology

To create pPRSK29 (*F25B3.3*::FLAG::PAB-1), 4 kb of the F25B3.3 promoter upstream of the predicted ATG start was amplified using the following primers: Dp-5 (5'-GTC AAC TAG TGT ATG ATT CCT CG-3') and Dp-3 (5'-TCG GGG TAC CTA TCG TCG TCG TCG TCG ATG CCG TCT TCA CGA-3'). The predicted ATG start of *F25B3.3 *was replaced with an Asp718 site in the 3' primer. This PCR fragment was cloned into pCR2.1-TOPO (Invitrogen, Carlsbad, California, USA) to generate pPRSK29.1. pPRSK29.1 was digested with *Bam*H1 and Asp718 to obtain the promoter fragment. pPRSK9 (*myo3*::FLAG::PAB-1) [11] was digested with Asp718 and *Sac*I to obtain the FLAG::PAB-1 fragment. pBluescript SK was digested with *Sac*I and *Bam*HI, and a threeway ligation was performed to obtain pPRSK29 (*F25B3.3*::FLAG::PAB-1).

### Transgenic generation

pPRSK29 (60 ng/μl) was co-injected with pTG99 (*sur-5*::GFP, 20 ng/μl) using standard injection protocols [106]. The resulting transgenic array was integrated using a Stratalinker (Stratagene) at 300 Joules/m^2 ^[107] (Shohei Mitani, personal communication). GFP reporters were selected at random from a subset of plasmids received from the Promoterome project [108]. Microparticle bombardment was conducted as described [5].

### Generating synchronized populations of L2 larvae for mRNA-tagging

Strains were grown to 'starvation' (that is, all dauer larvae) on ten 60 mm nematode growth media plates at 25°C. Half of each 60 mm plate was split into four pieces and placed on a 150 mm 8P plate [109] inoculated with the *E*. *coli *strain Na22. The resultant twenty 8P plates were incubated at 25°C until a majority of the food was depleted and most animals were gravid adults (a 'line' of worms is usually found at the retreating edge of the bacteria). The worms were removed from the plates with ice-cold M9 buffer (22 mM KH_2_PO_4_, 22 mM Na_2_HPO_4_, 85 mM NaCl, 1 mM MgSO_4_) and collected by centrifugation. Washes were repeated until the supernatant was clear of bacteria. A sucrose float (30 ml ice cold M9 buffer, 20 ml cold 70% sucrose) was performed to create an axenic nematode suspension. Animals were washed twice in ice-cold M9 buffer, then resuspended in 75 ml bleach solution (15 ml Chlorox, 3.75 ml 10 N NaOH, 56.25 ml water). Worms were transferred to a 125 ml glass beaker with a stir bar and incubated for 5-6 minutes while stirring rapidly (solution turns a dark yellow when nearing completion). When a majority of adults burst, the solution was passed through a 53 μm nylon mesh (Fisher #08670201, Pittsburgh, Pennsylvania, USA) to separate intact embryos from worm carcasses. Embryos were harvested by centrifugation and washed at least three times with M9 buffer. Embryos were resuspended in RT M9 buffer and incubated on a nutator for 12-16 hours at 20°C to allow L1 larvae to hatch and arrest.

Arrested L1 larvae were collected by centrifugation. Animals were resuspended in 1 ml RT M9 buffer and split equally over six 150 mm 8P plates. L1s were grown at 20°C for 22-25 hours to reach mid-L2, as shown by the appearance of the post-deirid sensory organ (approximately 80%) [1]. L2s (approximately 0.3-1 ml) were harvested from 8P plates and sucrose floated as above. Worms were resuspended in 30 ml cold M9.

### mRNA-tagging

Methods are identical to those previously described [11] with the following modifications. Synchronized L2 larvae were resuspended in 2-3 ml homogenization buffer (HB; 50 mM HEPES, pH 7.6; 150 mM NaCl; 10 mM MgCl_2_; 1 mM EGTA, pH 8.0; 15 mM EDTA, pH 8.0; 0.6 mg/ml Heparin; 10% glycerol) and passed through a French press at 6,000 psi. Total RNA was isolated from 100 μl of lysate. An amount of lysate equivalent to 200 μg total RNA was used for co-immunoprecipitation. Following co-immunoprecipitation, beads were washed three times by brief treatment with 2 ml low-salt homogenization buffer (LSHB; 20 mM HEPES, pH 7.6; 25 mM NaCl; 1 mM EGTA, pH 8.0; 1 mM EDTA, pH 8.0; 0.6 mg/ml Heparin; 10% glycerol). Beads were then washed three time for 30 minutes in 2 ml LSHB. The LSHB treatment substantially reduced nonspecific RNA binding to the agarose beads (data not shown). Elution and mRNA extraction were performed as described [11] (see detailed protocol in Additional data file 20).

### Isolation of RNA from embryonic neurons for MAPCeL analysis

In the MAPCeL method, GFP cells are isolated by FACS for microarray analysis. Primary cultures of embryonic cells were prepared [12] from a transgenic line expressing GFP throughout the nervous system, NW1229 (*evIs111*, *F25B3.3*::GFP) [47] (J Culotti, personal communication). After 24 hour in culture, GFP-labeled neurons were obtained by FACS and total RNA isolated as described [5,110]. Muscle profiling data used in Figures 4 and 7 were obtained by MAPCeL of embryonic muscle cells after 24 hours in culture (M24 dataset) (RMF, DMM, unpublished data). The top 50 enriched genes in this dataset were selected on the basis of statistical rank.

### RNA amplification and microarray data analysis

A *C. elegans *Affymetrix chip was used for all microarray experiments [111]. For mRNA-tagging experiments, 25 ng of co-immunoprecipitated RNA was amplified and labeled as previously described [5]. Larval pan-neural (*F25B3.3*::FLAG::PAB-1) profiles were obtained in triplicate. Four independent larval A-class motor neuron (*unc-4*::3XFLAG::PAB-1) profiles were obtained. Reference profiles were generated from low levels of non-specifically bound RNA obtained from mock immunoprecipitations of synchronized populations of wild type (N2) L2 larvae. Five independent reference datasets were obtained. Total RNA (100 ng) was amplified and labeled for the MAPCeL sample, *F25B3.3*::GFP, isolated in triplicate. A previously obtained profile of total RNA isolated from all viable embryonic cells in culture was used as a MAPCeL reference [5].

Hybridization intensities for each experiment were scaled by reference to a global average signal from the same array (Additional data files 25 and 26) and normalized by robust multi-array analysis (RMA; Additional data files 27 and 28). We identified transcripts in two categories: EGs, or transcripts that are reliably detected in a given sample; and enriched genes, or transcripts with intensity values that are significantly higher than reference samples. EGs were estimated for the mRNA-tagging samples as follows. Expressed transcripts in the *F25B3.3*::FLAG::PAB-1 (larval pan-neural) and the *unc4*::3XFLAG::PAB-1 (larval A-class motor neurons) were initially identified on the basis of a 'present' call in a majority (for example, two-thirds) of experiments as determined by Affymetrix MAS 5.0. In this approach, genes are called 'absent' and, therefore, excluded when the mismatch (MM) value exceeds the perfect match (PM) intensity for a given gene. This analysis initially identified 8,084 'present' transcripts in the larval pan-neural sample and 7,578 transcripts in the larval A-class motor neuron sample (Additional data file 21). These lists, however, are likely to include mRNAs that are non-specifically bound to the anti-FLAG sepharose beads at low levels relative to *bona fide *neuronal transcripts (see above). We reasoned that transcripts included in the experimental samples that are actually derived from this non-specific pool should be generally detected in the reference sample at higher intensity values. Therefore, to exclude these non-specific mRNAs from the list of predicted neuronal genes, the average RMA-normalized intensity for each transcript in the reference sample was subtracted from the RMA value of the corresponding gene in the experimental sample. Transcripts with resultant positive values were considered EGs whereas transcripts with negative values after this operation were removed. In a final adjustment, a limited number of transcripts that are detected as neuronally enriched (see below) but not scored as present by MAS 5.0 were restored to the lists. This treatment identified 4,033 EGs in the larval pan-neural dataset and 3,320 EGs in the larval A-class motor neuron profile (Additional data file 13). EGs (7,953) for the MAPCeL embryonic pan-neural dataset were identified as previously described (Additional data file 12) [5]. Our treatment is relatively stringent as it is likely to exclude at least some transcripts that may be ubiquitously expressed (for example, 'housekeeping' genes) or potentially more highly expressed in another tissue relative to the nervous system. This prediction is consistent with the finding that approximately 20% (509/2,422; Additional data file 15) of transcripts identified in independent microarray experiments as highly enriched in GMIc (GMI plus the genes common to all three groups) remain in the list of larval pan-neural EGs (Additional data file 13). In contrast, 48% (1,172/2,422; Additional data file 15) of transcripts enriched in these other tissues are included in the list of 6,342 EGs in the larval reference dataset (Additional data file 13).

The data discussed in this publication have been deposited in NCBI's Gene Expression Omnibus [112-114] and are accessible through GEO series accession number GSE8004 (embryonic pan-neural, larval pan-neural, larval A-class) and GSE8159 (embryonic A-class).

To detect neuronally enriched transcripts, RMA-normalized intensities for experimental versus reference samples were statistically analyzed using Significance Analysis of Microarrays software (SAM) [115]. A two-class unpaired analysis of the data was performed to identify genes that differ by ≥ 1.5-fold from the reference at a FDR of <1% for the larval pan-neural, embryonic pan-neural, and larval A-class motor neuron datasets (Additional data file 1). These genes were considered significantly enriched.

RMA normalized intensity values for all datasets were imported into GeneSpring GX 7.3 (Agilent Technologies, Santa Clara, California, USA) to generate the line graphs shown in Figures 4 and 7. Each experimental dataset was paired to its corresponding reference dataset for these diagrams.

### Annotation of datasets

We utilized Perl scripts and hand annotation to identify all known neuronally expressed *C. elegans *transcripts (WormBase Release 146 (WS146)). First, WormMart was used to identify all transcripts with expression patterns. This list was filtered for genes represented on the Affymetrix microarray. For genes that have multiple spots on the microarray, only one representative spot was kept in the list (3,044). Genes with expression patterns with no spatial information or exclusive to males were eliminated (2,837). Each gene was then placed into two categories based on its known expression pattern - neural (1,612) versus non-neural (1,225) - using the following criteria. We used a Perl script ('keyword_search.pl', Additional data file 22) to search descriptions of 2,837 genes with known expression patterns for genes with defined neural expression. To reduce the number of false positives identified, we first searched under the term 'cell group', which provides simple, but clear, spatial expression information. Using this strategy, the majority of neuronally expressed genes were separated from the full dataset. Several genes in WormBase, however, had no cell group, or contained insufficient data in the cell group description to determine neural expression. Therefore, WormBase was also searched for terms associated with neuronal expression. This list was hand-annotated to ensure its validity (for a full list of search terms, see Additional data file 23).

### Hypergeometric calculations

Overlap statistics were calculated using web-based software designed by Jim Lund (University of Kentucky) [116]. The number of genes in the genome was set at 18,666 (total number of genes represented on the *C. elegans *Affymetrix array). When using this calculation, a representation factor below 1.0 indicates under-representation, while a value above 1.0 indicates over-representation.

### Microscopy and identification of GFP-expressing cells

GFP-expressing animals were visualized by differential interference contrast (DIC) and epifluorescence microscopy using either a Zeiss Axioplan or Axiovert compound microscope. Digital images were recorded with CCD cameras (ORCA I, ORCA ER, Hammatsu Corporation, Bridgewater, NJ, USA).

### Identification of mouse homologs of uncharacterized conserved *C. elegans *pan-neural genes described in the Allen Brain Atlas

Twenty-six mouse homologs of the 27 uncharacterized conserved *C. elegans *genes (Additional data file 9) found in both embryonic and larval pan-neural enriched datasets were identified in Ensembl [117]. Mouse homolog gene names were then used to query the Allen Brain Atlas [118] for expression in the mouse brain. A gene was scored as 'expressed in the brain' if it had an intensity value of 10 or higher (normalized scale 0-100) in at least one brain region on the summary graph interface. The one exception was 1500041B16Rik, which did not have a summary graph; expression in the brain in this case was confirmed by direct visualization of the *in situ *photographs available in the Brain Atlas.

### *C. elegans *interactome

Genes enriched in both the larval and embryonic pan-neural datasets were used to seed the *C. elegans *interactome [67,119]. The map was trimmed to exclude genes with one interacting partner. The initial dataset consisted of 711 genes (Additional data file 10), of which 17% (124) were listed in the Interactome database. One large cluster of 34 interactors was identified and contains 17 proteins from the original seed. The additional 17 genes were categorized as enriched, expressed, or not present in the pan-neural datasets. Genes were assigned to categories based on known or predicted functions in *C. elegans *or other organisms.

## Additional data files

The following additional data are available with the online version of this paper. Additional data file 1 lists enriched genes from the larval pan-neural (LP), embryonic pan-neural (EP), larval A-class (LA), and embryonic A-class (EA) microarray datasets. Additional data file 2 is a master annotation file of all spots on the *C. elegans *Affymetrix microarray (based on WormBase releases WS140 and WS146). Additional data file 3 is a master annotation file of all genes represented on the *C. elegans *Affymetrix microarray (based on WormBase releases WS140 and WS146). Additional data file 4 lists LP, EP, LA, and EA enriched datasets categorized into gene families. Additional data file 5 is a comparison of the LP and LA enriched transcripts to the 1,926 GMI enriched genes found on the *C. elegans *Affymetrix chip [13]. Additional data file 6 lists the 50 top-ranked muscle enriched genes (RMF, DMM, unpublished data). Additional data file 7 lists LP enriched genes with known expression patterns from Figure 3b. Additional data file 8 includes representative scatter plots and R^2 ^values for pairwise combinations of the EP and LA datasets. Additional data file 9 lists 27 uncharacterized human homologues in the LP and EP datasets, and indicates mouse homolog and expression in mouse brain. Additional data file 10 shows provides comparisons of all enriched datasets (LP versus EP; LP versus LA; EP versus EA; LA versus EA) shown in Figure 8. Additional data file 11 provides comparisons of all enriched datasets versus a MAPCeL profile of chemosensory neurons (AFD/AWB) [8] shown in Figure 8. Additional data file 12 lists EGs from the embryonic EP, EA, and embryonic reference (ER) datasets. Additional data file 13 lists EGs from the LP, LA, and larval reference (LR) datasets. Additional data file 14 list 11,868 unique transcripts identified by all of our microarray experiments. Additional data file 15 provides a comparison of the GMIc dataset to the LP EG and LR EG datasets. Additional data file 16 lists the approximately 1,600 EGs without EST confirmation plus a subset enriched in the LP, EP, EA, and LA neural datasets.

Additional data file 17 is a complete list of GFP expression patterns. Additional data file 18 is a description of the dauer pathway genes identified in our LP and EP enriched datasets. Additional data file 19 lists the 162 common A-class enriched genes categorized into gene families. Additional data file 20 provides the mRNA-tagging bench protocol. Additional data file 21 lists the genes identified by MAS5.0 as present in at least two-thirds of LP replicates and three-quarters of LA replicates. The RMA normalized intensity values for these genes, plus those from the LR dataset, are included. Additional data file 22 is a Perl script used to search WormBase expression data (keyword_search.pl). Additional data file 23 list the keywords used to search WormBase expression data. Additional data file 24 lists enriched genes from all datasets (LP, EP, LA, EA) with uncharacterized human homologues. Additional data file 25 lists the MAS5.0 intensities for the EP, LP, and LA datasets. Additional data file 26 lists the MAS5.0 intensities for the LR dataset. Additional data file 27 lists the RMA intensities for the LP, LA, and LR datasets. Additional data file 28 lists the RMA intensities for the EP and ER datasets. Additional data file 29 is a comparison of the EP and LP enriched datasets to the list of candidate neural genes containing a presumptive pan-neural *cis*-regulatory element [48].

## Supplementary Material

Additional data file 1 Enriched genes from the larval pan-neural (LP), embryonic pan-neural (EP), larval A-class (LA), and embryonic A-class (EA) microarray datasets. Click here for file

Additional data file 2 Master annotation file of all spots on the *C. elegans *Affymetrix microarray (based on WormBase releases WS140 and WS146). Click here for file

Additional data file 3 Master annotation file of all genes represented on the *C. elegans *Affymetrix microarray (based on WormBase releases WS140 and WS146). Click here for file

Additional data file 4 Larval pan-neural (LP), embryonic pan-neural (EP), larval A-class (LA), and embryonic A-class (EA) enriched datasets categorized into gene families. Click here for file

Additional data file 5 Comparison of the larval pan-neural (LP) and larval A-class (LA) enriched transcripts to the 1,926 GMI enriched genes found on the *C. elegans *Affymetrix chip [13]. Click here for file

Additional data file 6 Fifty top-ranked muscle enriched genes. Click here for file

Additional data file 7 Larval pan-neural enriched genes with known expression patterns from Figure 3b. Click here for file

Additional data file 8 Representative scatter plots and R^2 ^values for pairwise combinations of the embryonic pan-neural (EP) and larval A-class (LA) datasets. Click here for file

Additional data file 9 Twenty-seven uncharacterized human homologues in the larval pan-neural (LP) and embryonic pan-neural (EP) datasets, and mouse homologs and expression in mouse brain. Click here for file

Additional data file 10 Comparisons of all enriched datasets (larval pan-neural (LP) versus embryonic pan-neural (EP); LP versus larval A-class (LA); EP versus embryonic A-class (EA); LA versus EA) shown in Figure 8. Click here for file

Additional data file 11 Comparisons of all enriched datasets versus a MAPCeL profile of chemosensory neurons (AFD/AWB) [8] shown in Figure 8. Click here for file

Additional data file 12 EGs from the embryonic pan-neural (EP), embryonic A-class (EA), and embryonic reference (ER) datasets. Click here for file

Additional data file 13 EGs from the larval pan-neural (LP), larval A-class (LA), and larval reference (LR) datasets. Click here for file

Additional data file 14 11,868 unique transcripts identified by all of our microarray experiments. Click here for file

Additional data file 15 Comparison of the GMIc dataset to the larval pan-neural (LP) EG and larval reference (LR) EG datasets. Click here for file

Additional data file 16 The approximately 1,600 EGs without EST confirmation plus a subset enriched in the larval pan-neural (LP), embryonic pan-neural (EP), embryonic A-class (EA), and larval A-class (LA) neural datasets. Click here for file

Additional data file 17 Complete list of GFP expression patterns. Click here for file

Additional data file 18 Description of the dauer pathway genes identified in our larval pan-neural (LP) and embryonic pan-neural (EP) enriched datasets. Click here for file

Additional data file 19 The 162 common A-class enriched genes categorized into gene families. Click here for file

Additional data file 20 mRNA-tagging bench protocol. Click here for file

Additional data file 21 The RMA normalized intensity values for these genes, plus those from the larval reference (LR) dataset, are included. Click here for file

Additional data file 22 Perl script used to search WormBase expression data (keyword_search.pl). Click here for file

Additional data file 23 Keywords used to search WormBase expression data. Click here for file

Additional data file 24 Enriched genes from all datasets with uncharacterized human homologues. Click here for file

Additional data file 25 MAS5.0 intensities for the embryonic pan-neural (EP), larval pan-neural (LP), and larval A-class (LA) datasets. Click here for file

Additional data file 26 MAS5.0 intensities for the larval reference (LR) dataset. Click here for file

Additional data file 27 RMA intensities for the larval pan-neural (LP), larval A-class (LA), and larval reference (LR) datasets. Click here for file

Additional data file 28 RMA intensities for the embryonic pan-neural (EP) and embryonic reference (ER) datasets. Click here for file

Additional data file 29 Comparison of the embryonic and larval pan-neural enriched datasets to the list of candidate neural genes containing a presumptive pan-neural *cis*-regulatory element [48]. Click here for file
