# Supplementary material for: Cell-specific microarray profiling experiments reveal a comprehensive picture of gene expression in the C. elegans nervous system
Source: Genome Biol. 2007 Jul 5;8(7):R135. doi: 10.1186/gb-2007-8-7-r135 (PMC2323220; doi:10.1186/gb-2007-8-7-r135)
Supplement: Additional data file 8 — Representative scatter plots and R2 values for pairwise combinations of the embryonic pan-neural (EP) and larval A-class (LA) datasets. [file gb-2007-8-7-r135-S8.ppt]

## Slide 1
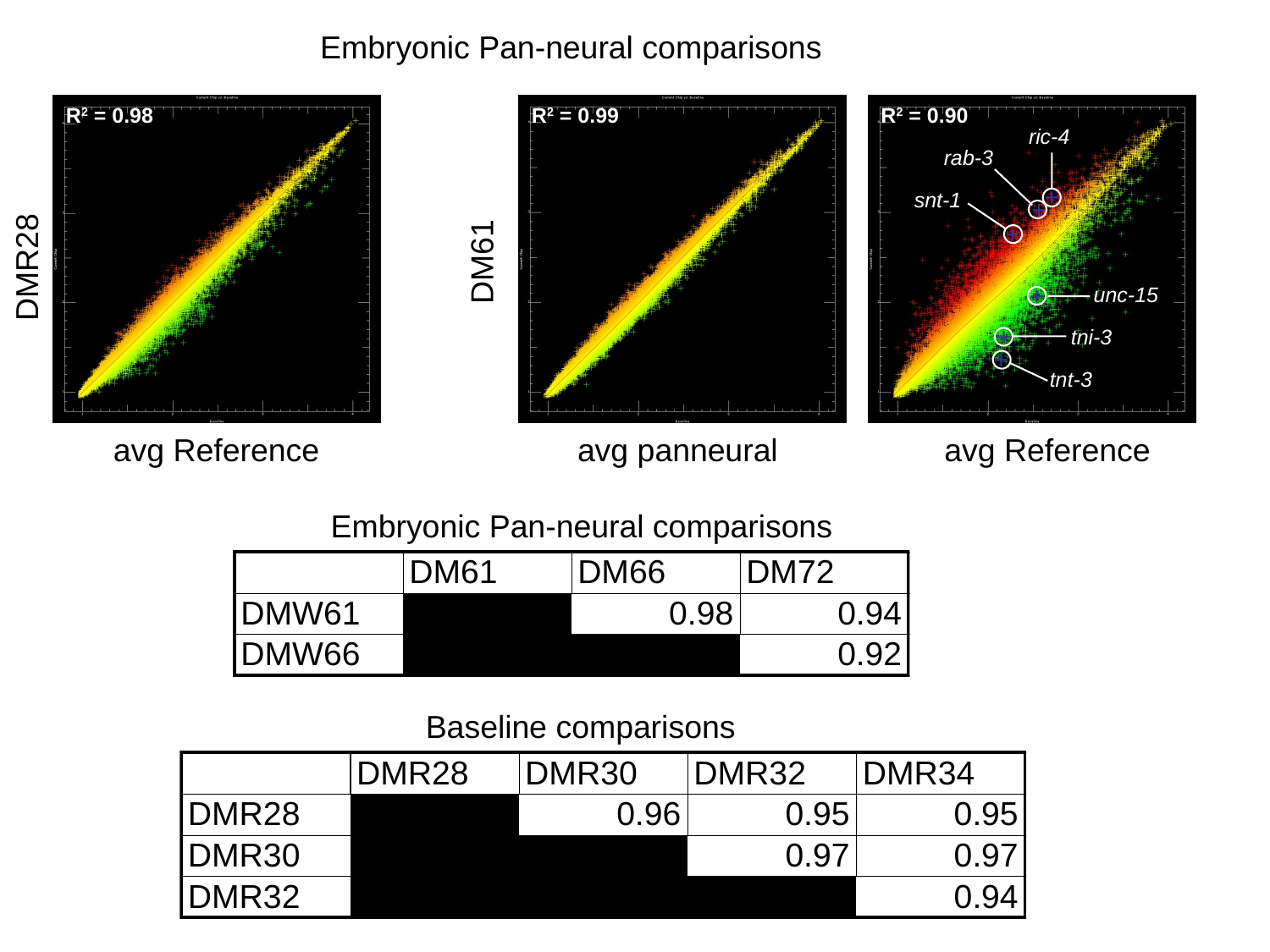

Embryonic Pan-neural comparisons
R2 = 0.98
R2 = 0.99
ric-4
rab-3
+
snt-1
+
+
+
unc-15
+
tni-3
+
tnt-3
R2 = 0.90
DM61
DMR28
avg Reference
avg panneural
avg Reference
Embryonic Pan-neural comparisons
Baseline comparisons

## Slide 2
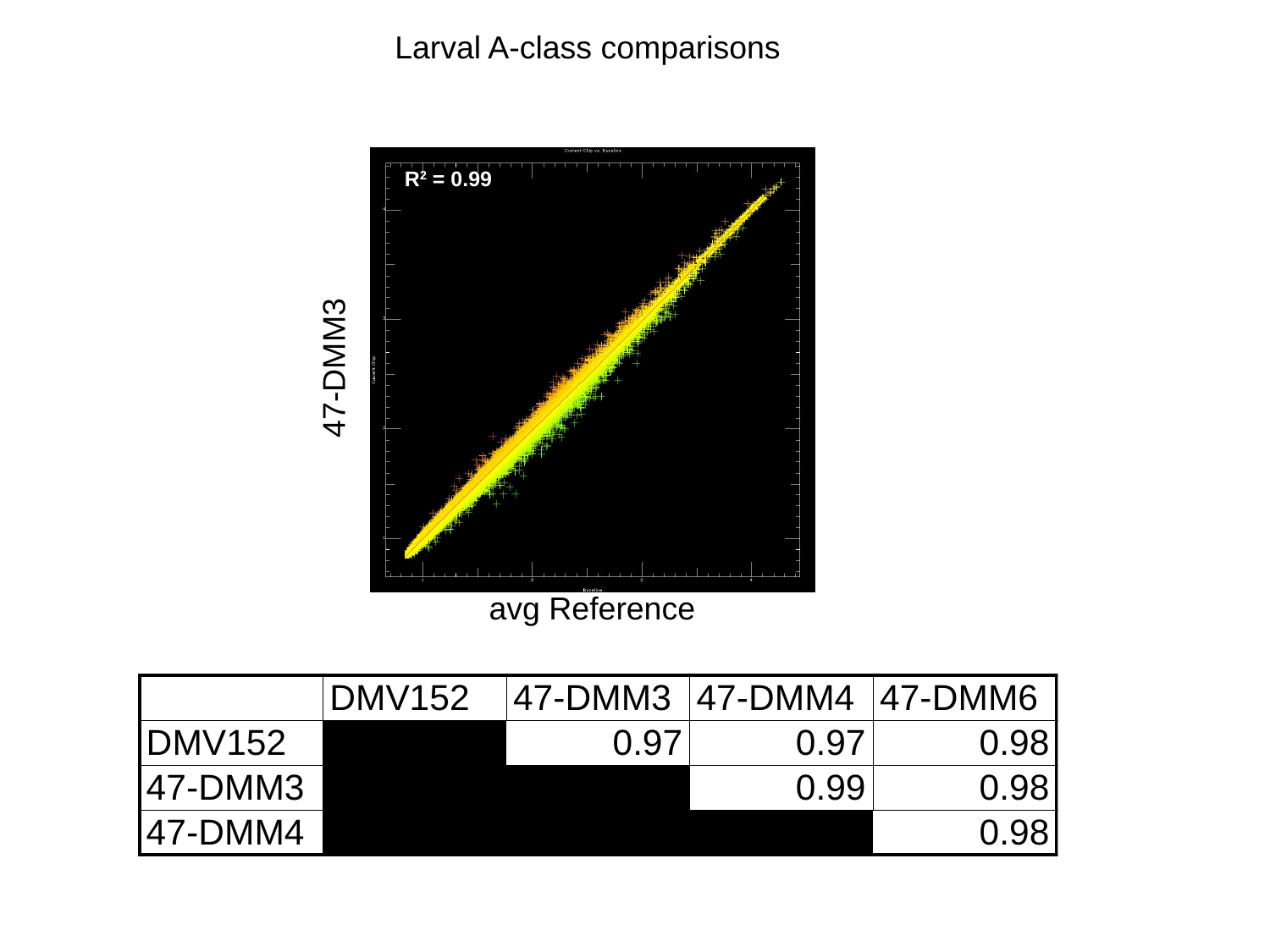

Larval A-class comparisons
R2 = 0.99
47-DMM3
avg Reference
