# Supplementary material for: Cell-specific microarray profiling experiments reveal a comprehensive picture of gene expression in the C. elegans nervous system
Source: Genome Biol. 2007 Jul 5;8(7):R135. doi: 10.1186/gb-2007-8-7-r135 (PMC2323220; doi:10.1186/gb-2007-8-7-r135)
Supplement: Additional data file 17 — Complete list of GFP expression patterns. [file gb-2007-8-7-r135-S17.pdf]

Cosmid Name	Common Name	Protein	Pan-neural		A-class		GFP expression: Neurons	GFP expression: Other cells
			Emb	Larval	Emb	Larval		
C01G6.4		Predicted E3 ubiquitin ligase	1.8				All ventral cord motor neurons, head and tail neurons, touch neurons	Intestine, vulval muscle, pharyngeal muscle, anal depressor, body muscle
VF11C1L.1	*ppk-3*	PIP kinase	1.8				All ventral cord motor neurons, head and tail neurons, PLN	Pharyngeal muscle, vulval muscle, anal depressor, hypodermis
C25D7.8		novel	1.9		2.9		Weak in head and tail neurons, ventral cord	Pharynx, sheath cells, distal tip cell
F08G12.1			3.0		2.0		Bright in head neurons, few tail neurons. weak in all ventral cord motor neurons. Touch neurons, PDE.	Intestine, head muscle, pharyngeal muscle, hypodermis, distal tip cell
M79.1	*abl-1*	Abelson kinase	2.3		2.4		All ventral cord motor neurons, head and tail neurons	
Y71D11A.5		Ligand-gated ion channel	2.1	1.8	2.5		DA, DB, DD, VA, VB, VD, AS head and tail neurons	Body muscle, head muscle, pharyngeal muscle
F25G6.4	*acr-15*	Acetylcholine receptor		4.9			All VNC motor neuron classes, except DD, head and tail neurons	
T27A1.6[1]	*mab-9*	Transcription factor		1.7	2.3		DA, DB, DD, VD, AS	
F39G3.8[1]	*tig-2*	TGF-β		1.8	1.8		VA, VB, DA, DB, AS touch neurons	Body muscle, pharyngeal neurons
T19C4.5[1]		novel		2.0	2.2			
CC4.2[1,3]	*nlp-15*	neuropeptide		6.5	2.6		DD, head and tail neurons	Body muscle, pharyngeal muscle
C18H9.7	*rpy-1*	rapsyn		2.7	1.7		DA, VD, AS, VB, DB	Body muscle
C04E12.7		Phospholipid scramblase		3.2		1.8	All ventral cord motor neurons, head and tail neurons, touch neurons	Body muscle, anal depressor, sphincter muscle? Excretory gland cells?
F36A2.4[1,4]	*twk-30*	K+ channel		2.1	3.5	5.1	All ventral cord neurons	
Y71H9A.3	*sto-4*	stomatin		3.0		1.6	Posterior ventral cord motor neurons (VA, VB, DB, AS), head and tail neurons, touch neurons	

F29G6.2[1]		novel		3.2	1.9	1.6	All neurons	
C44B11.3	*mec-12*	Alpha-tubulin		5.9		1.9	L2 -- DA, VA, VB, VD, bright in touch neurons, head and tail neurons<br>L3 -- all ventral cord motor neurons	
T23D8.2[1]	*tsp-7*	tetraspanin		3.5	4.5	4.8	All ventral cord neurons, head and tail neurons	
T05C12.2[1]	*acr-14*	Acetylcholine receptor		1.5	2.8	3.1	DA, VB, AS, DB, DD, HSN, VC4&5, AIY, head neurons	Muscle, intestine
F33D4.3[1,2]	*flp-13*	neuropeptide		7.1	8.7	7.9	I5, ASE, ASG, BAG, DD, M3, M5, head neurons	
C11D2.6[1]	*nca-1*	Ca++ channel		2.3	4.0	2.2	DA, DB, VA, VB, head and tail neurons	
E03D2.2[1,3]	*nlp-9*	neuropeptide		3.1	6.3	2.5	VA, head neurons	Intestine
F55C12.4[1]		novel		3.5	1.9	2.1	DA, VB, DB, VD, DD, bright in AS, also head and tail neurons	
F43C9.4[1,5]	*mig-13*	CUB domain		1.8	3.0	2.8	Bright in anterior ventral cord neurons, weak and mosaic in posterior ventral cord.	Pharyngeal/intestinal valves, hypodermis
F39B2.8		Predicted membrane protein	1.7	3.5	2.0	2.1	DA, DB, VA, VB, DD, VD, head neurons	Pharyngeal muscle
K02E10.8[1]	*syg-1*	Ig domain	1.8	1.5	1.8	1.8	DA, DB, DD, VA, VB, VD, AS, head neurons	Head muscle
ZC21.2[1]	*trp-1*	Ca++ channel	1.9	2.2	2.1	1.9	DA, DB, VA, VB	
Y47D3B.2a[1,6]	*nlp-21*	neuropeptide	3.9	8.3	5.3	3.7	DA, DB, VA, VB, AS, head neurons	Body muscle, intestine
F09C3.2		phosphtase	1.9	2.7	5.1	1.7	DA, DB, VA, VB, VD	Intestine, hypodermis
T27E9.9		Ligand-gated ion channel	2.3	4.0	3.6	3.1	DA, DB, VA, VB, VC, touch neurons, head and tail neurons	
Y34D9B.1	*mig-1*	Frizzled-like				1.6	All ventral cord motor neurons, head neurons, touch neurons	Intestine, anal depressor, head muscle

black boxes = not detected, red boxes = enriched, yellow boxes = expressed gene (EG)

1. Fox, RM et al., 2005
2. Kim, K and Li, C, 2004
3. Li, C et al., 1999
4. Salkoff, L et al., 2001
5. Sym, M et al., 1999
6. Nathoo, AN et al., 2001
