# Supplementary material for: Cell-specific microarray profiling experiments reveal a comprehensive picture of gene expression in the C. elegans nervous system
Source: Genome Biol. 2007 Jul 5;8(7):R135. doi: 10.1186/gb-2007-8-7-r135 (PMC2323220; doi:10.1186/gb-2007-8-7-r135)
Supplement: Additional data file 20 — mRNA-tagging bench protocol. [file gb-2007-8-7-r135-S20.doc]

# PAB-1 Protocol

# (Stephen Von Stetina, Joseph Watson, David Miller)

Express epitope tagged Poly-A Binding Protein (PAB-1) in cells of interest

Formaldehyde fix and lyse synchronized L2 larvae

Immunoprecipitate PAB-1 bound mRNA using FLAG antibody

Amplify and biotinylate mRNA using modified Eberwine approach

Fragment and hybridize to the *C. elegans* Affy array

# Materials Needed

| Reagent Name | Vendor | Catalog # |
| --- | --- | --- |
| HEPES | Sigma | H-3375, 100 g |
| Sodium chloride | Sigma | S-7653, 250 g |
| Magnesium chloride | Sigma | M-2670, 100 g |
| Heparin | Sigma | H-3393, 100 g |
| Glycerol | Sigma | G-5516, 500 ml |
| DTT | Fisher | BP172, 5 g |
| Glycine | Sigma | G-7403, 100 g |
| EGTA | Sigma | E-3906, 10 g |
| EDTA | Fisher | BP118-500, 500 g |
| Ribonucleoside vanadyl complexes, 200 mM | Sigma | R-3380, 10 ml |
| Anti-FLAG EZView Agarose beads | Sigma | F-2426, 1 ml |
| rRNasin | Promega | N-2515, 10000U |
| Complete, Mini, EDTA freeProtease inhibitor tablets | Roche | 1 836 170, 25 tablets |

**Solutions**

**Use DEPC-H_2_O, baked spatulas, beakers, and stir bars, and powders found in RNase-free cabinet for all solutions. Store solutions in baked 100 ml bottles.**

DEPC-H_2_O

# Fill 4L Flask in RNase-free cabinet with 3.5L of Milli-Q water. In hood, add 3.5 ml DEPC (final concentration is 0.1%). Leave stirring at RT o/n. Autoclave the following day.

# ****Use diluted HCl or NaOH made with DEPC-H_2_O to pH solutions

# 0.5 M HEPES 5M NaCl 1M MgCl_2_ 0.5M EDTA 0.1M EGTA

# 11.92 g HEPES 29.22g NaCl 20.3g MgCl_2_ 14.61g EDTA 3.8g EGTA

# 75 ml DEPC-H_2_O 75 ml DEPC-H_2_O 75 ml DEPC-H_2_O 75 ml DEPC-H_2_O 75 ml DEPC-H_2_O

pH to 7.6 bring up to 100 ml bring up to 100 ml pH to 8.0 pH to 8.0

bring up to 100 ml autoclave autoclave bring up to 100 ml bring up to 100 ml

autoclave autoclave autoclave

Homogenization Buffer (HB) Low salt homogenization buffer

Amounts Final Conc. Amounts Final Conc.

30 ml 5 M NaCl 150 mM 5 ml 5 M NaCl 25 mM

100 ml 0.5M HEPES, pH 7.6 50 mM 40 ml 0.5M HEPES, pH 7.6 20 mM

10 ml 1M MgCl_2_ 10 mM 10 ml 0.1M EGTA 1 mM

10 ml 0.1M EGTA 1 mM 2 ml 0.5M EDTA 1 mM

30 ml 0.5M EDTA 15 mM 600 mg Heparin 0.6 mg/ml

600 mg Heparin 0.6 mg/ml 100 ml glycerol 10%

100 ml glycerol 10% 843 ml DEPC-H_2_O

720 ml DEPC-H_2_O DEPC-treat the solution (1 ml DEPC).

DEPC-treat the solution (1 ml DEPC). Autoclave. **Store at 4°C**.

Autoclave. **Store at 4°C**.

20% Paraformaldehyde 0.1M Glycine Elution Buffer 1M Tris

Weigh out 400-500 mg 0.75 g Glycine Amounts Final Conc. 6.06 g THAM

Resuspend in 4.5Xml weight 75 ml DEPC-H_2_O 0.5 ml 1M Tris, pH 8.0 50 mM 30 ml DEPC-H_2_O

(in mg) ul 5mM NaOH pH to 3.5 0.2 ml 0.5M EDTA, pH 8.0 10 mM pH to 8.0

Incubate at 65°C, mixing bring up to 100 ml 1.3 ml 10% SDS 1.3% bring up to 50 ml

occasionally, until dissolved autoclave bring up to 10 ml

1M DTT

Dissolve 1.54 g DTT in 10 ml DEPC-H_2_O. Filter sterilize (use syringe filters). Store in 500 ul aliquots at -20°C

10% SDS Dissolve 5 g SDS in 50 ml DEPC-H_2_O. Filter Sterilize. Store at RT.

Ribonucleoside vanadyl complexes

It’s a dark frozen liquid. Thaw slowly on ice, then aliquot (1.5 ml) into RNase-free microfuge tubes. Store at -80°C.

Anti-FLAG beads

Mix well. Aliquot 150 ul into 1.5 ml RNase-free microfuge tubes (to get ~100 ul packed beads). Store at -20°C

10 ml Oakridge polycarbonate centrifuge tubes

Fill tubes with RNase-AWAY, then mix on Nutator o/n. Rinse with DEPC-H_2_O 3X, then fill with DEPC-H_2_O and nutate 3-4 hr. Air dry on RNase-free bench, then cap them and store in drawer.

Worm Growth and Synchronization

1. For every IP you wish to do (every independent sample for the microarray), place chunked out agar pieces of starved worms from ten 60mm NGM plates onto twenty 150mm 8P plates (1/2 NGM plate/ 8P plate).
2. Grow at 25°C until plates are full of gravid adults (usually 3-4 days).
3. Wash worms off plates with cold M9 and put into 50 ml conical tubes on ice.
4. Spin in centrifuge (using 7.5 rotor) at 3000 rpm for 5 min at 4°C.
5. Remove supernatant. Pool all worms into one 50 ml tube. Wash with M9 until supernatant is clear.
6. Add 30 ml cold M9 buffer plus 20 ml 70% sucrose. Mix well. Centrifuge immediately as above.
7. Remove floating worms with 6mm glass pasteur pipet (cut tip off with diamond-tipped pen, use flame to smooth edge). Wash 2X with cold M9. (You should have 1-5 ml packed worms).
8. Bleach worms to release eggs
   1. Make 75 ml bleach solution (15 ml chlorox, 3.75 ml 10N NaOH, 56.25 ml water)
   2. Resuspend worms in 50 ml bleach solution.
   3. Transfer to 125 ml beaker with stir bar. Add 25 ml more bleach solution.
   4. Stir rapidly with foil covering beaker for 4-6 min (begin checking by placing a drop on a glass slide and observing the number of adults which have burst). Solution turns deep yellow when most worms are lysed.
   5. Filter through 53 um spectra nylon mesh (Fisher #08-670-201) in an embroidery hoop. Hoop is placed on a 1L beaker to collect filtrate.
   6. Split filtrate into four 50 ml conical tubes, add M9 up to 50 ml.
   7. Spin in clinical centrifuge at 2500 rpm for 2.5 min.
   8. Quickly remove supernatant. Pool eggs, wash 3X more with M9 (or until bleach smell gone).
9. Resuspend eggs in 50 ml RT M9 buffer. Place one drop of eggs onto 60 mm NGM plate to check for viability. Split resuspended eggs equally into two 50 ml conical tubes. Place tubes on nutator in 20°C incubator. Let eggs hatch o/n (usu. 12-15 hours)
10. Check plate to see if most of the eggs hatched. If so, continue on.
11. Centrifuge conical tubes with L1 larvae at 3000 rpm for 5 min at 4°C.
12. You should have 300 ul-1 ml packed L1s. Resuspend L1s in each tube with 1 ml RT M9 buffer.
13. For each tube: use glass pasteur pipet (with end broken off, see step 7) to put 1 ml L1s onto 3 150 mm 8P plates in small drops. Wash tube with 0.5 ml RT M9 buffer and distrubute over the same 3 plates. Let liquid soak in (leave uncovered in plate-pouring hood -- usually dry in 15-30 min).
14. Incubate for ~22 h at 20°C for L2s. (Check for postdeirid division using DIC optics).

Postdeirid

From Sulston and Horvitz, 1977.

**Obtain L2 lysates**.

(For every 1 batch of L2s to press, prepare 35 ml homogenization buffer [HB])

1. Add 1 protease inhibitor cocktail tablet to 35 ml HB. Nutate at 4°C until dissolved.
2. After tablet has dissolved, add:
   1. 35 ul RNase-free 1M DTT to HB (Final: 1 mM).
   2. 43.75 ul rRNasin (Final: 400U/8 ml).
   3. 1.4 ml 200 mM vanadyl ribonucleoside complex (Final: 8 mM) to HB. (Keep nutating at 4°C so the vanadyl doesn’t collect in the bottom of the tube.)
3. When the post-deirid divisions have occurred in >80% animals, wash L2s off plates with cold M9 buffer. Wash and sucrose as above (worm growth & synch. steps 3-7).
4. You should have 300 ul-1 ml L2s. Resuspend in 30 ml M9 buffer.
5. Add 750 ul 20% paraformaldehyde (Final: 0.5% paraformaldehyde) to fix worms.
6. Incubate at 4°C on nutator for 1 hour.
7. Wash worms with 50 ml M9 buffer. Use clinical centrifuge to pellet worms at 2500 rpm for 2.5 min.
8. Wash worms with 25 ml HB.
9. Resuspend worms in 2-3 ml HB. (if packed worms were 1 ml or greater, use 3 ml—otherwise use 2-2.5 ml). Transfer to 15 ml conical tube.
10. Place worms on ice.
11. French press worms (use Wente lab ThermoSpectronic French press with our mini-cell [FA-003] and spacer [FA-010]).
    1. You will need sterile transfer pipets, goggles, and a 500 ml beaker of ice.
    2. Make sure to remove the extensions from the support rods.
    3. Set up mini-cell as directed in manufacturer’s directions (Figure 1):
       1. Place vacuum grease over O-rings.
       2. Screw flow valve assembly and sample outlet tube in appropriate holes.
       3. Open flow valve assembly, then push piston into body.
       4. Push closure plug into body.


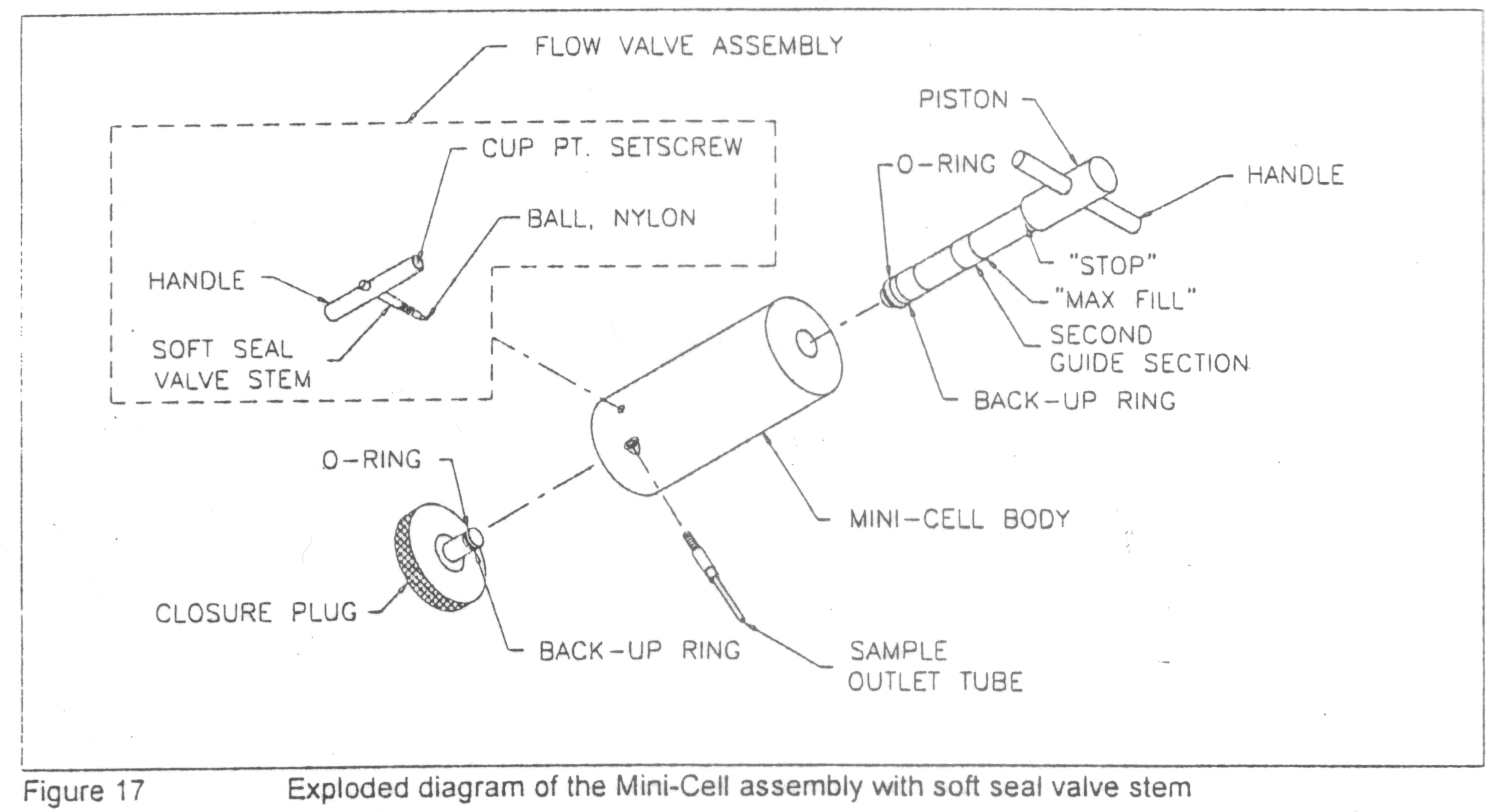


**Figure 1**

- 1. Add sample to the cell.
     1. Turn cell upside down, remove closure plug, place on tripod.
     2. Pull piston out until you see the max-fill line.
     3. Add sample to cell body using transfer pipet.
     4. Push piston in until level of liquid just below outlet hole.
     5. Make sure flow valve is OPEN. Push closure plug into body, making sure to hold piston in place.
     6. Close flow valve.
     7. Turn cell right-side up and place on spacer.
     8. Attach cell clamp, making sure that the piston arms are perpendicular to the thumb screws.
     9. Place tubing coming from outlet into 15 ml conical tube placed in beaker of ice.
  2. Press the worms.
     1. Make sure pressure knob is all the way to the left. Turn press on.
     2. Set pressure to 340 (6000 psi).
     3. Move handle from down to med.
     4. Once top of piston touches ‘roof’, check to see that pressure is still at 340. (if not, make necessary adjustments). Slowly open flow valve assembly until worm lysate comes out—you want 1 drop/sec.
     5. When the piston is completely down, move handle back to down.
     6. Remove cell, reload, press again.
     7. Remove cell, reload, press again.
     8. If pressing another sample, rinse press with DEPC-H_2_O.
        1. Set up cell as if you are loading another sample.
        2. Squirt DEPC-H_2_O into chamber.
        3. Make sure flow valve is OPEN, then push closure plug into body.
        4. Position outlet tubing so that water will drain into ice.
        5. Simply push the piston down to squirt the water out.
        6. Repeat 3X.
        7. Follow above directions to press another sample.
     9. After all samples have been pressed, return to lowest position, turn pressure knob to the left and turn press off.

1. Homogenize the worm lysate 30 times using baked glass homogenizer.
2. Transfer lysate to 10 ml Oak Ridge polycarbonate centrifuge tubes (NalgeNunc #3118-0010).
3. Spin in centrifuge
   1. Use Beckman rotor JS-13, with both 15 ml and 10 ml adapters. 15 ml adapter is blue rubber, 10 ml is white plastic (stored in cold room; adapters that are weight matched are numbered the same).
   2. Make sure tubes that will be across from each other are balanced!!!! Add HB to tubes to match weight. (be within 0.05 g of each other)
   3. Spin for 6 min at 5000 rpm (~4000 x*g*)at 4°C.
4. Remove supernatant. Place in new centrifuge tube.
5. Spin
   1. Use Beckman rotor JS-13 with 10 and 15 ml adapters.
   2. Make sure tubes are balanced to within 0.02 g.
   3. Spin for 20 min at 11,750 rpm (~20,000 x*g*) at 4°C.
6. Transfer lysate into 15 ml conical tube using transfer pipet.
7. Perform Trizol extraction on 100 ul of lysate to determine concentration of total RNA.
   1. Add 400 ul Trizol (4X vol) to pooled eluate. Incubate 5 min at RT.
   2. Mix well, then add 100 ul (1X vol) chloroform. Incubate 10 min at RT.
   3. Mix, then spin for 15 min at 14000 rpm at 4°C.
   4. Remove aqueous layer to new tube.
   5. Extract with equal volume chloroform. Spin for 10 min at 14000 rpm at 4°C.
   6. Remove aqueous layer to new tube.
   7. Add equal volume isopropanol. Incubate RT 10 min.
   8. Spin for 25 min at 14000 rpm at 4°C.
   9. Wash pellet 2X with 70% EtOH.
   10. AIR DRY PELLET! Resuspend in 32 ul DEPC-H_2_O.
   11. Dilute 1:50, take A260 reading to determine concentration for later use.
   12. Example: if concentration is 1 ug/ul, then in 30 ul there is 30 ug total RNA, which came from 100 ul lysate. Therefore 100 ul lysate = 30 ug total RNA.
8. Aliquot remaining lysate into microfuge tubes tubes in 0.5 ml aliquots.
9. Flash freeze in liquid nitrogen; store at -80 °C until ready to do immunoprecipiation.

**Immunoprecipitation.**

1. Prepare homogenization buffers (amounts vary depending on # of IPs).
   1. Need 4 ml HB to equilibrate beads and < 1 ml for IP (see step 5 below).
   2. Need 12 ml low-salt HB to wash beads post-IP.
2. Prepare anti-FLAG M2 agarose beads (all spins at 3500 rpm for 5 min at 4°C)
   1. Remove 100 ul aliquot of beads (at –20°C) for each IP.
   2. Wash 2X with 1 ml RNase-free 0.1M glycine, pH 3.5.
   3. Wash 4X with 1 ml HB.
3. Store at 4°C until ready to use.
4. Determine amount of lysate which corresponds to 200 ug total RNA (see Obtain L2 lysates, step 18, above).
   1. Example: if in 100 ul lysate there is 30 ug total RNA, then for 200 ug total RNA you would need 660 ul lysate.
5. Add lysate to 100 ul prepared anti-FLAG M2 agarose beads.
   1. add HB to 1 ml, then add 10 ul rRNasin, 8 ul vanadyl ribonucleoside complex,
6. Incubate for 2 hrs at 4°C on nutator.
7. Spin beads down at 3500 rpm, 4°C, 3 min.
8. Remove supernatant. Add 1 ml low-salt HB, transfer to RNase-free 2 ml microfuge tube. Add 1 ml low-salt HB to rinse 1.5 ml IP tube, then combine with beads in 2 ml tube.
9. Mix well, spin as above.
10. Wash beads 2X with 2 ml low-salt HB Buffer, as above.
11. Wash beads 3X with 2 ml low-salt HB, placing on nutator at 4°C for 30 minutes.
12. After final wash, remove all but 500 ul HB
    1. transfer remaining 500 ul HB + beads to 1.5 ml microfuge tube. Rinse 2 ml tube with 500 ul HB then add to beads in 1.5 ml tube. Spin down, remove supernatant.
13. Elute:
    1. Add 125 ul elution buffer (preheated to 65°C) and 10 ul rRNasin to beads.
    2. Tape microfuge tubes to glass hybridization tube. Rotate for 30 min in hybridization oven set to 65°C.
    3. Spin down beads (3500 rpm for 5 min at 4°C).
    4. Remove eluate to 1.5 ml microfuge tube on ice.
    5. Repeat elution, then pool eluates. (If residual beads present, spin down again, then remove supernatant for extraction).
14. Extract RNA
    1. Add 1 ml Trizol (4X vol) to pooled eluate. Incubate 5 min at RT.
    2. Mix well, then add 250 ul (1X vol) chloroform. Incubate 10 min at RT.
    3. Mix, then spin for 15 min at 14000 rpm at 4°C.
    4. Remove aqueous layer to new tube.
    5. Extract with equal volume chloroform. Spin for 10 min at 14000 rpm at 4°C.
    6. Remove aqueous layer to new tube.
15. Precipitate RNA
    1. Add equal volume isopropanol. Incubate RT 10 min.
    2. Spin for 25 min at 14000 rpm at 4°C.
    3. Wash pellet 2X with 70% EtOH.
    4. AIR DRY PELLET!
16. Resuspend IPed mRNA in 12 ul DEPC-H_2_O.
17. Take a spec reading:
    1. Add 2 ul RNA to 98 ul DEPC-H_2_O (1:50 dilution).
    2. Take A260 reading (I read same sample twice).
    3. Determine concentration
       1. Avg. A260 x 40 ug/ml x 50 (dil. factor)/1000 = amt RNA (ug/ul)
18. If necessary, dilute a portion to 100 ng/ul. Remove 3 ul for bioanalysis.
19. Dilute a portion to 25 ng/ul. Add 1 ul (25ng) RNA to 9 ul DEPC-H_2_O in a 0.5 ml RNase-free microfuge tubes—use for amplification.
20. Store RNA at –80^o^C.
